# Diazotrophic Bacteria *Pantoea dispersa* and *Enterobacter asburiae* Promote Sugarcane Growth by Inducing Nitrogen Uptake and Defense-Related Gene Expression

**DOI:** 10.3389/fmicb.2020.600417

**Published:** 2021-01-12

**Authors:** Pratiksha Singh, Rajesh Kumar Singh, Hai-Bi Li, Dao-Jun Guo, Anjney Sharma, Prakash Lakshmanan, Mukesh K. Malviya, Xiu-Peng Song, Manoj K. Solanki, Krishan K. Verma, Li-Tao Yang, Yang-Rui Li

**Affiliations:** ^1^Key Laboratory of Sugarcane Biotechnology and Genetic Improvement (Guangxi), Ministry of Agriculture, Sugarcane Research Center, Chinese Academy of Agricultural Sciences, Nanning, China; ^2^Guangxi Key Laboratory of Sugarcane Genetic Improvement, Sugarcane Research Institute, Guangxi Academy of Agricultural Sciences, Nanning, China; ^3^Guangxi Key Laboratory of Crop Genetic Improvement and Biotechnology, Nanning, China; ^4^State Key Laboratory of Conservation and Utilization of Subtropical Agro-Bio Resources, College of Agriculture, Guangxi University, Nanning, China; ^5^Guangxi South Subtropical Agricultural Science Research Institute, Guangxi Academy of Agricultural Sciences, Nanning, China; ^6^Interdisciplinary Center for Agriculture Green Development in Yangtze River Basin, College of Resources and Environment, Southwest University, Chongqing, China; ^7^Queensland Alliance for Agriculture and Food Innovation, The University of Queensland, St Lucia, QLD, Australia; ^8^Department of Food Quality and Safety, The Volcani Center, Institute for Post-Harvest and Food Sciences, Agricultural Research Organization, Rishon LeZion, Israel

**Keywords:** antagonism, colonization, defense-related genes expression, nitrogen fixation, ^15^N isotope, PGPR, sugarcane

## Abstract

Sugarcane is a major crop in tropical and subtropical regions of the world. In China, the application of large amounts of nitrogen (N) fertilizer to boost sugarcane yield is commonplace, but it causes substantial environmental damages, particularly soil, and water pollution. Certain rhizosphere microbes are known to be beneficial for sugarcane production, but much of the sugarcane rhizosphere microflora remains unknown. We have isolated several sugarcane rhizosphere bacteria, and 27 of them were examined for N-fixation, plant growth promotion, and antifungal activity. 16S rRNA gene sequencing was used to identify these strains. Among the isolates, several strains were found to have a relatively high activity of nitrogenase and ACC deaminase, the enzyme that reduces ethylene production in plants. These strains were found to possess *nifH* and *acdS* genes associated with N-fixation and ethylene production, respectively. Two of these strains, *Pantoea dispersa*-AA7 and *Enterobacter asburiae*-BY4 showed maximum plant growth promotion (PGP) and nitrogenase activity, and thus they were selected for detailed analysis. The results show that they colonize different sugarcane tissues, use various growth substrates (carbon and nitrogen), and tolerate various stress conditions (pH and osmotic stress). The positive effect of AA7 and BY4 strains on *nifH* and stress-related gene (*SuCAT*, *SuSOD*, *SuPAL*, *SuCHI*, and *SuGLU*) expression and the induction of defense-related processes in two sugarcane varieties, GT11 and GXB9, showed their potential for stress amelioration and PGP. Both bacterial strains increased several sugarcane physiological parameters. i.e., plant height, shoot weight, root weight, leaf area, chlorophyll content, and photosynthesis, in plants grown under greenhouse conditions. The ability of rhizobacteria on N-fixing in sugarcane was also confirmed by a ^15^N isotope-dilution study, and the estimate indicates a contribution of 21–35% of plant nitrogen by rhizobacterial biological N fixation (BNF). This is the first report of sugarcane growth promotion by N-fixing rhizobacteria *P. dispersa* and *E. asburiae* strains. Both strains could be used as biofertilizer for sugarcane to minimize nitrogen fertilizer use and better disease management.

## Introduction

Sustainable production of food to feed the growing population remains a major challenge. This may be achieved by expanding the area of agriculture, pest and disease management, increasing soil fertility, agriculture intensification, and improving crop varieties. Sugarcane is an important bioenergy and sugar crop globally and is also a source of raw materials for various industrial products. China is the third-largest sugarcane-producing country in the world with Guangxi province producing ∼60% of the sugar produced in the country (Li et al., 2016; FAOSTAT, 2020). Sugarcane, a long-duration high-biomass crop, has a relatively high demand for nitrogen (N) to sustain its high productivity. In China, the rate of nitrogen (N) fertilizer application in commercial sugarcane production, though showing a decreasing trend, is reported to be between 500 and 700 kg N ha^–1^, much higher than in other countries (Robinson et al., 2011). The application of higher N fertilizer contributes to the soil, groundwater, and air pollution, and higher cost of crop production (Zhu and Chen, 2002; Yang et al., 2020). Moreover, sugarcane diseases such as smut, red rot, wilt, and ratoon stunt disease also cause extensive yield loss in many countries (Viswanathan and Rao, 2011).

Diazotrophic microbes comprise groups of free-living organisms capable of enzymatically reducing the atmospheric N into bioavailable N for plant, and thus, they enhance crop growth and yield (Bashan and Bashan, 2010; Cassán et al., 2015). The N-fixation mechanism of diazotrophic bacteria is thus beneficial for plant growth and development, and some of them can also provide induced systemic resistance (ISR) to phytopathogens and some abiotic stresses (Olanrewaju et al., 2017). Biological N fixation provides 30–80% of sugarcane N requirements in certain production regions (Taulé et al., 2012; Urquiaga et al., 2012; Li et al., 2013). Diazotrophic bacteria also improve soil biology and soil structure and composition (Gopalakrishnan et al., 2017). Nitrogen-fixing rhizobacterial inoculation has increased N fixation ability and improved growth in sugarcane plants (Lin et al., 2012; Li et al., 2017; Singh et al., 2020). Several genera of Enterobacteriaceae, Erwiniaceae, and other related families such as *Enterobacter* sp., *Klebsiella* sp., *Serratia* sp., and *Pantoea* sp. were involved in N-fixation and growth promotion in sugarcane and other crops worldwide (Madhaiyan et al., 2010; Mehnaz et al., 2010; Taghavi et al., 2010; Taulé et al., 2012; Witzel et al., 2012). With promising leads in reducing diseases and nutrition, substantial efforts are now underway in identifying and characterizing beneficial diazotrophic rhizosphere microorganisms from different crops (Vannier et al., 2019).

Biosynthesis of defense-related molecules occurs during the interaction between diazotrophic plant growth-promoting rhizobacteria (DPGPR) and host plants (Singh et al., 2020). The increased levels of stress-protective proteins give the host plant a greater chance of survival under adverse conditions (Jan et al., 2011; Kumar and Verma, 2018). The molecular and biochemical bases of rhizobacteria-derived pathogen tolerance, however, remain unclear. Some evidence implicates rhizobacterial colonization-induced changes in reactive oxygen species (ROS) and secondary product metabolism in disease tolerance (Sahran and Nehra, 2011; Hassan et al., 2014; Rashid and Chung, 2017). The mechanism of ROS detoxification differs with plant species and age and the occurrence and severity of abiotic and biotic stresses plants experience (Hodges et al., 1996). Plant growth-promoting rhizobacteria (PGPR) induce plant defense-related genes such as phenylalanine ammonia-lyase (PAL), catalase (CAT), ascorbate peroxidase (APX), peroxidase (POD), and superoxide dismutase (SOD), which may assist the plant to protect from or reduce the impact of pathogens attacks (Cassán et al., 2014; Fukami et al., 2017; Bhattacharyya et al., 2020).

The goal of this research was to isolate and identify the N-fixing and plant growth-promoting (PGP) potential of rhizobacterial strains in sugarcane. The *Enterobacter* and *Pantoea* genera are among the most phenotypically varied in Enterobacteriaceae and Erwiniaceae, and both live in a range of habitats and coexist with diverse rhizospheric and endophytic associations with plants (Quecine et al., 2012; Chen et al., 2017). *Pantoea* and *Enterobacter* strains show wide environmental and plant host adaptation, and thus, they are receiving considerable attention for their PGP traits with the ultimate objective of identifying new valuable DPGPR strains for using as biofertilizers for sustainable agriculture (Walterson and Stavrinides, 2015). Hence, our specific objectives were (i) to isolate and characterize diazotrophic bacteria associated with rhizosphere soil of sugarcane grown in Nanning, Guangxi, China; (ii) investigate them for nitrogen fixation, PGP traits, and antagonistic activity against sugarcane pathogens; (iii) selection and assessment of most promising strains to expand our molecular understanding of a beneficial plant–microbe interaction using a number of analytical tools and approaches such as confocal microscopy, gene expression, N-fixation-associated metabolic changes, and ^15^N isotope dilution; and (iv) evaluation of selected rhizobacteria for plant growth parameters of two sugarcane varieties under different conditions.

## Materials and Methods

Four sugarcane (*Saccharum* spp. interspecific hybrids) varieties (GT11, GT29, GXB9, and ROC22) used for this research were obtained from the same sugarcane field located in Guangxi University Experimental Farm in Nanning, Guangxi, South China. It has subtropical weather with annual temperatures ranging between 8 and 33°C. It is located between 22°49′1.21′ N latitude and 108°21′59.55′′ E longitude, with a 79.51 m elevation. Twenty healthy 6 month-old sugarcane plants from each variety were collected simple randomly from four different places of the sugarcane field (size 72 × 25 m) in April 2015. The soil adhered to the roots was separated manually by gently removing it from roots (Barillot et al., 2013), and after removing the debris, the soil was filtered through a 2 mm sieve, and the filtered soil fraction was stored at 4°C for further use. These fine soil samples (filtrate) were processed within 24 h for rhizobacteria isolation and soil physicochemical analysis.

### Isolation of Nitrogen-Fixing Rhizobacteria

Nitrogen-fixing bacteria (NFB) were isolated by using four different growth media: Ashbey medium (Hi-Media), Yeast Mannitol Agar (Hi-Media), JNFb medium (Baldani et al., 1992), and LGI medium (Baldani, 1984; Supplementary Table S1). All selected enrichment media contained nutrients that allowed the growth of NFB. To isolate NFB, 10 gm of soil samples was mixed with 90 mL of saline water and kept at 32 ± 2°C for 60 min in an orbital shaker set at 120 rpm. After incubation, this saline soil mixture was spread on the abovementioned media and incubated for 2–3 days at 32 ± 2°C. A total of 350 bacterial colonies were selected and purified for further studies. All pure cultures were stored in 30% glycerol at −80°C.

### *In vitro* Plant Growth-Promoting (PGP) Traits

All isolated strains were studied for their PGP traits such as phosphate solubilization, siderophore, ammonia, HCN, and indole-3-acetic acid (IAA) production using selective growth media following standard screening procedures.

For the phosphate (P) solubilization test, all selected DPGPR were inoculated on Pikovskayas agar (Hi-media) medium, incubated for 3–5 days at 32 ± 2°C, and observed for the development of a clear zone surrounding the bacterial colony (Pikovskaya, 1948). All DPGPR were tested for siderophore production using the Chrome azurol S (CAS) agar medium (Schwyn and Neilands, 1987). In short, the pure bacterial colonies were inoculated on the CAS medium and kept for 3 days at 32 ± 2°C. The development of the orange zone around the bacterial colonies confirmed siderophore production by DPGPR. The ammonia production by nitrogen-fixing strains was determined by inoculating freshly grown strains in peptone water (10 mL) and incubating at 32 ± 2°C for 2–3 days. After incubation, 0.5 mL of Nessler’s reagent was added to the bacterial peptone water and the production of yellow color was used to confirm ammonia production (Dey et al., 2004). Hydrogen cyanide (HCN) production of selected rhizobacterial strains was measured according to Lorck (1948). Briefly, bacterial strains were inoculated in 15 mL Luria–Bertani (LB) broth containing glycine (4.4 g L^–1^) in a test tube and a Whatman filter paper (No. 1) soaked in 1% picric acid solution was hanged in the test tube and sealed with parafilm wax. These cultures were incubated at 32 ± 2°C for 5–6 days, and the change in filter paper color from orange to red established HCN production. Indole-3-acetic acid (IAA) production was determined using the colorimetric method in the presence and absence of L-tryptophan as described previously by Gordon and Weber (1952). The overnight grown pure colony of each DPGPR was inoculated in LB broth and kept for 3 days at 32 ± 2°C. After incubation, bacterial cultures were centrifuged at 6,000 rpm for 15 min and 2 mL of the supernatant was mixed with orthophosphoric acid (2 drops) and Salkowski’s reagent (4 mL). IAA was measured spectrophotometry (UV-160 A, Shimadzu, Japan) at 530 nm.

### Screening for Antifungal Activity

The antifungal activity of bacterial isolates was tested against two different sugarcane fungal pathogen strains (*Sporisorium scitamineum* causing sugarcane smut disease and *Ceratocystis paradoxa* causing sugarcane pineapple disease) using a dual-culture method with potato dextrose agar (PDA): Luria–Bertani (LB) (1:1) agar plates incubated at 28 ± 2°C for 5 days or until the mycelium was completely grown up in the control plate. The rhizobacterial strains displaying inhibition of fungal pathogen growth were considered as significant biocontrol strains.

### 1-Aminocyclopropane-1-Carboxylic Deaminase (ACCD) Activity

The capacity of all selected strains to use 1-aminocyclopropane-1-carboxylic acid (ACC) as a sole source of nitrogen was evaluated using Dworkin and Foster (DF) salt minimal medium containing ACC (3 mM) (Penrose and Glick, 2003). For this analysis, fresh pure cultures grown in LB broth were used. The bacterial broth was centrifuged at 10,000 rpm for 5–6 min at 5°C, and the pelleted bacterial was spotted on medium containing ACC. 1-Aminocyclopropane-1-carboxylic deaminase activity was quantified by measuring the production of α-ketobutyrate (Honma and Shimomura, 1978), and the activity was presented as nmol α-ketobutyrate mg^–1^ protein h^–1^.

### Nitrogen Fixation Through Acetylene Reduction Assay (ARA)

Nitrogenase activity of all strains was studied by the ARA method (Hardy et al., 1968). A pure rhizobacterial colony was inoculated on 10 mL semi-solid NFb medium (Supplementary Table S1) in a test tube and incubated at 32 ± 2°C for 36–48 h. Then, under sterile conditions air was removed from the tubes and replaced with acetylene gas (5 mL), and the test tube was kept for another 24 h at 32 ± 2°C. At the end of the incubation, 0.5 mL of headspace gas was carefully extracted from each tube and analyzed in a GC-17A gas chromatograph (Shimadzu, Japan) with DB-1,701 column (Agilent, Santa Clara, United States) set using the flame ionization detector (FID) at 80°C and the injector at 110°C, with 35 mL min^–1^ flow rate of carrier gas. The quantity of ethylene (C_2_ H_4_) produced by each strain was calculated and presented as nmol C_2_H_4_ produced mg protein^–1^ h^–1^.

### DNA Extraction, Amplification, and Sequencing of Nitrogen-Fixing Rhizobacteria

Sugarcane nitrogen-fixing rhizobacterial strain identification was done by partial 16S rRNA gene sequencing. All isolates were grown in LB broth for 24–36 h on a gyratory shaker kept at 160 rpm at 32 ± 2°C. The genomic DNA of these samples was extracted with a DNA isolation kit (CWBIO, Beijing, China). The DNA quality and purity were determined by gel electrophoresis (0.8% agarose; wt/vol) and quantified by Nanophotometer (Pearl, Implen-3,780). PCR amplification of the 16S rRNA gene was done by universal primer sets pA-F and pH-R (Supplementary Table S2; Edwards et al., 1989). All amplified PCR products were sequenced by Sangon Biotech (Shanghai, China).

### Phylogenetic Analysis

Identification of all strains and phylogenetic analysis based on 16S rRNA gene sequences were completed with reference sequences obtained from the NCBI GenBank database. ClustalW and BlastN search programs were used for sequence alignment through NCBI, and the closely related sequences were downloaded. The phylogenetic analysis based on 16S rRNA sequence was conducted with MEGAX for the 16S rRNA gene (Kumar et al., 2018) using the Neighbor-Joining method (Saitou and Nei, 1987). The genetic evolutionary distances were calculated by the number of differences method (Nei and Kumar, 2000), and the bootstrap test (1,000 replicates) was carried out as described by Felsenstein (1985).

### Amplification of *nifH* and *acdS* Genes

All rhizobacterial strains were screened for the presence of *nifH* and *acdS* genes using PCR with degenerate primer sets (Supplementary Table S2). The *acdS* gene amplification has been generally used for the identification of ACCD producing DPGPR. The PCR reaction was carried out in a 50 μL reaction volume for both *nifH* (Poly et al., 2001) and *acdS* (Li et al., 2011), and gene sequences were determined by direct sequencing of PCR products (Sangon Biotechnol Ltd., Shanghai, China). Gene sequences were verified using the BlastN search in the NCBI GenBank database.

### Phenotypic Microarray Assays Using BIOLOG^(R)^ Plates

Based on the various PGP traits, biocontrol, and nitrogenase activities, two most potent DPGP, strains BY4 and AA7, were selected for further studies. Carbon and nitrogen preferences and osmotic stress, and pH tolerance of BY4 and AA7 were analyzed by phenotypic assays. These analyses were conducted with BIOLOG Micro-Array^TM^ plates GENIII, PM3B, PM9, and PM10 (Biolog Inc., Hayward, CA). BIOLOG Micro-Array^TM^ plates include 96 wells, and each well comprises a different formulation to detect substrate utilization or sensitivity to stresses. Plates GENIII and PM3B were used to classify strains for their ability to utilize various carbon and nitrogen sources, whereas PM9 and PM10 plates are used for screening microbial tolerance to high salt concentrations and extreme pH. The name of 96 different substrates present in all four selected BIOLOG plates is presented in a Supplementary File (Supplementary Table S3). For this study, freshly grown cultures of BY4 and AA7 were inoculated in LB broth medium and incubated at 32 ± 2°C for 48–72 h then centrifuged and washed 3–4 times with autoclaved distilled water. The pellets were transferred to an inoculation fluid (IF), as advised by the BIOLOG^(R)^ protocol. Finally, 100 μL of bacterial suspension prepared with IF was transferred into 96 wells of GENIII, PM3B, PM9, and PM10 plates were incubated for 48 h at 32 ± 2°C, and purple color development confirmed the substrate utilization and stress tolerance of DPGPR.

### Monitoring of Bacterial Colonization in Sugarcane

Bacterial colonization in plant and rhizosphere was monitored using pPROBE-pTet^r^-TT plasmid expressing green fluorescent protein (GFP) provided by the Agriculture college, Guangxi University, Nanning, China.

#### Tagging of Bacterial Strains With GFP-pPROBE-pTet^r^-TT

The selected isolates (AA7 and BY4) were mixed with pPROBE-pTet^r^-TT at a 1:2 ratio in LB broth and incubated at 32 ± 2°C for 36–48 h in an orbital shaker set at 160 rpm. After the incubation, 100 μL aliquot of bacterial broth was spread on LB agar plates overnight to check the purity of tagged strains and also to confirm the tagging using confocal laser scanning microscopy (CLSM). Bacteria displaying green fluorescence upon ultraviolet light illumination were selected for further studies.

#### Bacterial Colonization Study Using GFP-Tagged pPROBE-pTet^r^-TT Rhizobacteria

The micro-propagated sugarcane plantlets (variety GT11) provided by Sugarcane Research Institute, Guangxi Academy of Agriculture Sciences, Nanning, China, were used for this experiment. The roots of *in vitro* plants were washed with autoclaved distilled water prior to bacterial inoculation. Three sugarcane plantlets were transferred to an autoclaved glass bottle containing MS liquid medium (50 mL). After 3–4 days of incubation at 30 ± 2°C in a growth chamber, plantlets were carefully transferred to another bottle containing tagged bacterial suspension with ∼ 2.0 × 10^5^ mL^–1^ cell count and placed in a growth chamber set at 30 ± 2°C with a 14 h photoperiod and 60 μ moL m^–2^ s^–1^ photon flux density. After 72–96 h of growth, plantlets were removed and washed with distilled water and examined for bacterial colonization using CLSM. Sugarcane root, stem, and leaf samples were cut into 1 cm-long pieces and mounted on the bridge slide with glycerol (10% v/v) and detected with CLSM at different emission rates on the intensity of autofluorescence UV light (Li et al., 2017) (Leica DMI 6,000, Mannheim, Germany).

### Scanning Electron Microscopy (SEM)

Bacterial colony morphology of BY4 and AA7 strains was studied according to the procedure described by Singh et al. (2013) using the Hitachi model SU8100 scanning electron microscope.

#### Response of Sugarcane Inoculated With AA7 and BY4 Isolates

##### Plant materials and experimental design

The effect of inoculating sugarcane with AA7 and BY4 isolates on plant growth and development was studied in two sugarcane varieties, GT11 and GXB9, grown in a greenhouse in the Agriculture College, Guangxi University, Nanning, China. Both sugarcane varieties were obtained from the Sugarcane Research Institute, Guangxi Academy of Agricultural Sciences, Nanning, China. The experiment was designed as a randomized block, with each block containing five replicates (pots) per treatment. There were three treatments: (1) no bacterial inoculation (incubation in sterile water: control), (2) inoculation with AA7 (*P. dispersa*), and (3) inoculation with BY4 (*E. asburiae*) in this experiment. A plastic pot (30 cm diameter and 40 cm deep) containing 15 kg soil and sand mixture (3:1 w/w) and having three treated plants were used as the experimental unit (replicate) for each treatment. Forty-five-day-old sugarcane plantlets were taken from the nursery, and roots were washed with flowing tap water to remove soil particles attached to the root surface. The roots were then inoculated with bacteria by immersing them in 1.0% carboxy-methyl cellulose (CMC) suspension containing rhizobacterial cells at 10^7^ CFU mL^–1^ for 30 min. Sugarcane plantlets with roots immersed in water without rhizobacterial cells were treated as control. Inoculated plants were potted and maintained in the greenhouse at > 80% relative humidity, 16/8 h light-dark cycle, and 30 ± 2°C ambient temperature for 90 days.

##### Plant sampling and analysis of physiological parameters, and pathogen defense-related enzymes and their gene expression

Plant height, shoot weight, root weight, leaf area, chlorophyll content, and photosynthesis of experimental plants were measured on 30, 60, and 90 days after inoculation (DAI). The middle portion of the youngest fully expanded leaf of each plant was used for photosynthesis measurements using a LI-6,800 compact portable photosynthesis system, Bluestem OS^TM^ version 1.3 (LI-COR Biosciences, Lincoln, Nebraska, United States). The activities of superoxide dismutase (SOD), phenylalanine ammonia-lyase (PAL), catalase (CAT), chitinase (CHI), and glucanase (GLU) in leaf and root tissues of sugarcane were determined at 30, 60, and 90 DAI, using the enzyme-linked immune sorbent assay (ELISA) kit (Wuhan Colorful Gene Biological Technology Co. Ltd, China) following the manufacturer’s directions.

The gene expression of *nifH*, *SuSOD*, *SuCAT*, *SuPAL*, *SuCHI*, and *SuGLU* was determined in sugarcane after bacterial inoculation (Supplementary Table S2). Leaf tissue from all treatments was sampled on 30 and 60 DAI, and total RNA was extracted with Trizol reagent (Tiangen, China) following the manufacturer’s instructions. RNA extract was treated with DNase I (Promega, United States), and then the first-strand cDNA was synthesized using the cDNA synthesis kit Prime-Script^TM^ RT Reagent Kit (TaKaRa, China) following the manufacturer’s protocols. qRT-PCR was performed with an SYBR Premix Ex Tap^TM^ II (TaKaRa, Japan) in a Bio-Rad RT-PCR (United States) machine as described earlier (Singh et al., 2020). A housekeeping gene glyceraldehyde 3-phosphate dehydrogenase (GAPDH) was used to normalize qRT-PCR data. The primer sequences used in this analysis are listed in Supplementary Table S2. The relative expression of all genes was calculated with a 2^–△^^△^^Ct^ procedure (Livak and Schmittgen, 2001).

##### Determination of nitrogen fixation using ^15^N isotope dilution technique

The analysis of nitrogen N content in the plant by ^15^N isotope dilution and ^15^N natural abundance methods are the most common approaches for quantifying N fixation in plants. Nitrogen fixation was measured in sugarcane tissues (root, stem, and leaf) inoculated with AA7 and BY4 strains.

Briefly, soil mixed with sand at a 1:3 ratio (w/w) was autoclaved in plastic bags at 121°C for 60 min, allowed to cool overnight, and autoclaved and cooled again. To the soil–sand mixture, 10 mg of ammonium sulfate−^15^N (10.12 atom percent ^15^N excess per kg soil wet weight was added (Shanghai Research Institute of Chemical Laboratory, China) and mixed for 2 weeks for a homogenous distribution of ^15^N. Finally, a soil–sand mixture with ^15^N was transferred to the pot and they were laid out following a randomized block design. Every block had three pots (replicates) from each treatment, and each pot had three plants from a single treatment. There were three treatments: (1) no bacterial inoculation (incubation in sterile water: control), (2) inoculation with AA7 (*P. dispersa*), and (3) inoculation with BY4 (*E. asburiae*) in this experiment. After 6 months of growth in a greenhouse, plants were removed carefully from the soil, the roots cleaned with distilled water to remove soil adhered to it, and they were separated into roots, stem, and leaf. Tissue samples were taken from each plant part, dried at 70°C till they reach a constant weight and ground to a fine powder, and filtered through a 0.5 mm sieve. The filtered powder fraction was analyzed for ^15^N enrichment by a K05 automatic Kjeldahl nitrogen analysis device (Sonnen Automated Analysis Ltd.) and an elementary analysis isotope ratio mass spectrometer (Thermo Fisher DeltaV) located at the Institute of Genetics and Physiology, Hebei Academy of Agriculture and Forestry Sciences, China. The contribution of N resulting from the air (Ndfa) was estimated by the following equation (Urquiaga et al., 1992).

Ndfa=100×[1-(atom%N15excessinoculatedplant/

atom%15Nexce6sscontrolplant)]

### Statistical Data Analysis

Statistical significance of experimental data was determined by analysis of variance followed by multiple comparisons based on Tukey’s HSD test. Mean values were used to calculate the standard error and statistical significance level calculated at *p* ≤ 0.05. All analyses were completed by SPSS software version 11.5. All PGP traits were measured in triplicates, and the results were presented as mean values. Venn diagram was prepared using Venny 2.1 software (Oliveros, 2007). Principal component analysis was done with OrigiPro 9.1 (2013). A heat map was prepared according to Babicki et al. (2016).

## Results

### Physicochemical Analysis of Soil

A total of 16 rhizosphere soil samples (four samples per variety) were collected from four sugarcane varieties i.e., GT11 (1), GXB9 (2), GT29 (3), and ROC22 (4). Soil samples of each variety were pooled to form a single combined sample, thus producing four separate variety-specific samples. The texture of the soil was medium loam with water content, pH, and electrical conductivity varying between 5.13 and 6.18%, 5.99 and 6.7, and 0.007 and 0.011 Sm^–1^, respectively. The N, phosphorus, and potassium contents ranged between 0.34 and 1.23, 0.40, and 0.46, and 13.29 and 14.37 g kg^–1^ (Supplementary Table S4). Soil samples did not show any deficiency for calcium, magnesium, and micronutrients (Supplementary Table S4).

### Isolation, Identification, and Characterization of Rhizobacteria With PGP Traits

**FIGURE 1:**
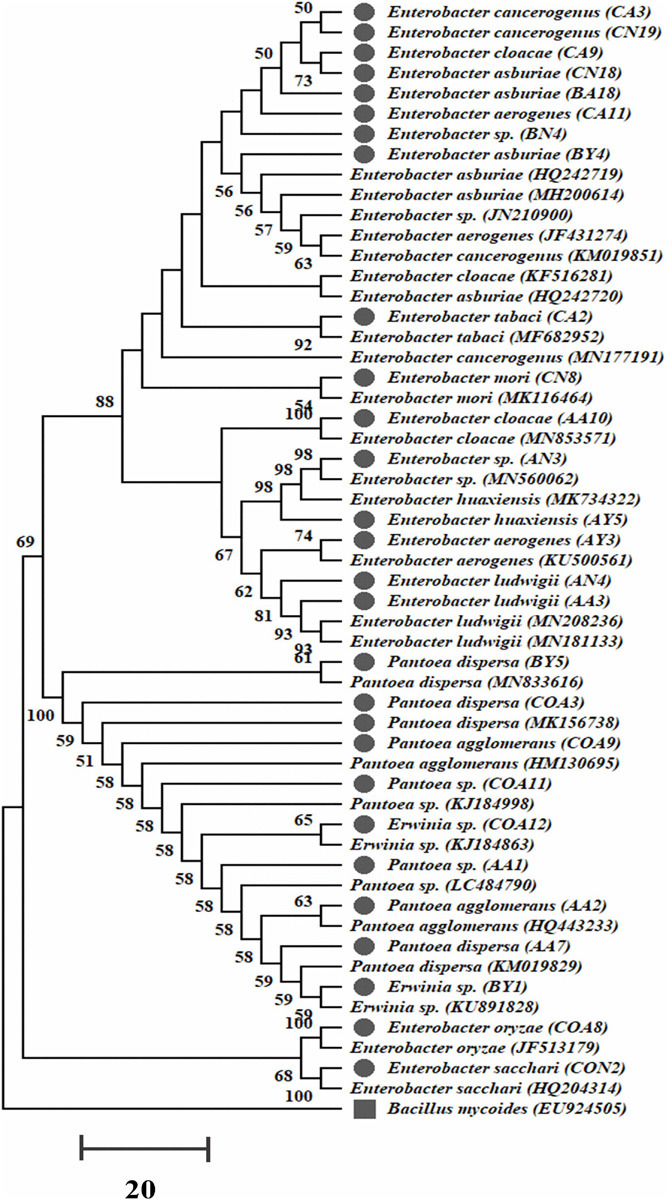
The 16S rRNA gene phylogenetic tree of 27 nitrogen-fixing PGP strains isolated from sugarcane and evolutionary distances were designed with the neighbor-joining method. Bootstrap values of 1,000 replications are designated as percent confidence values for specific branching. Scale bar denotes the number of variations per base position with *B. mycoides* as an outgroup.

A total of 350 rhizobacterial strains were isolated by using four different selective media from the sugarcane rhizosphere. Out of them, only 102 rhizobacteria showed various PGP traits, nitrogenase activity, and antifungal activity against the sugarcane pathogens. These 102 rhizobacteria were classified into five families after 16S rRNA gene sequencing, i.e., Pseudomonadaceae, Bacillaceae, Xanthomonadaceae, Enterobacteriaceae, and Erwiniaceae. Earlier, a few strains belonging to the family Enterobacteriaceae and Erwiniaceae were reported from sugarcane. In this study, we selected 27 strains of these two families after 16S rRNA gene sequencing for further analysis (Figure 1 and Supplementary Table S5). The 16S rRNA gene sequencing results revealed that all selected 27 strains belong to three different genera with eighteen strains of *Enterobacter* (*E. oryzae*, *E. sacchari*, *E. aerogenes*, *E. huaxiensis*, *E. ludwigii*, *E. cloacae*, *E. asburiae*, *E. tabaci*, *E. cancerogenus*, *E. mori*, and *E.* species), seven of *Pantoea* (*P*. *dispersa*, *P. agglomerans*, and *P*. *species*), and two of *Erwinia* sp. (Supplementary Table S5). All sequences of the rhizobacteria 16S rRNA gene were deposited to NCBI GenBank with accession numbers (MT557006–MT557032). The phylogenetic tree of all 27 strains was built, and it contained two major and four minor clades (Figure 1).

The P-solubilization was carried out for all N-fixing rhizobacterial strains by growing them on Pikovskaya’s medium. Results showed that 20 (74%) strains could solubilize a tricalcium phosphate and produced a halo zone on plate assay. Of these, thirteen strains exhibited a maximum zone of inhibition for the phosphate solubilization test (Table 1 and Supplementary Figure S1). Among the 27 strains tested, 22 strains (81%) produced an orange halo zone on the CAS agar plate showing siderophore production (Table 1 and Supplementary Figure S1). Sixteen (59%) out of 27 rhizobacterial strains produced ammonia, whereas only 8 (30%) strains produced HCN (Table 1 and Supplementary Figure S1). Indole-3-acetic acid production by the tested rhizobacteria ranged from 12.43 to 100.63 μg mL^–1^ without tryptophan in the medium, while the presence of tryptophan increases the range from 36.45 to 903.31 μg mL^–1^ with CN18 and AA10 strains corresponding to the minimum and maximum levels, respectively (Table 2).

**TABLE 1:** List of plant growth-promoting traits and antagonistic activity of rhizobacteria isolated from sugarcane.

**Strain code**	**Phosphate**	**Siderophore**	**Ammonia**	**HCN**	**ACC**	**Biocontrol activity**	
						***S. scitamineum***	***C. paradoxa***
AA1	+++	+++	++	−	+	+	−
AA2	+++	+++	++	−	+	+	+++
AA3	−	+++	+++	−	−	++	−
AA7	+++	++	++	+++	+	+++	+++
AA10	−	+++	++	+++	+	−	+++
AN3	++	+++	−	−	+	−	−
AN4	−	−	++	−	−	+	+++
AY3	+++	+++	−	−	−	++	+
AY5	++	+++	++	−	+	++	−
BA18	+++	+++	−	++	+	−	+++
BN4	+++	−	−	−	−	−	+ +
BY1	+++	−	−	+++	+	−	−
BY4	++	+++	++	+++	+	+++	+ ++
BY5	+++	−	++	−	−	++	+
CA2	+++	++	−	++	+	++	−
CA3	−	++	−	−	+	−	++
CA9	+++	++	−	−	+	−	++
CA11	−	++	+++	++	+	−	+++
CN8	+++	+++	++	−	+	+	++
CN18	++	−	+++	−	−	−	+++
CN19	−	+++	+++	+++	+	+	++
CoA3	++	+++	−	−	−	+	+
CoA8	+	+++	++	−	+	+	+
CoA9	+++	+++	−	−	+	−	+++
CoA11	+++	+++	−	−	−	−	+++
CoA12	−	+++	++	−	−	−	−
CoN2	++	+++	++	−	+	−	+

*Strength for all PGP traits: strong activity (+++); moderate activity (++); and low activity (+); no activity (−).*

**TABLE 2:** Quantitative analysis of rhizobacterial strains for indole-3-acetic acid production, acetylene reduction assay, and 1-aminocyclopropane-1-carboxylic deaminase activity.

**Strain code**	**IAA (μ g mL^–1^)**		**ARA (*n*moL C_2_H_4_ mg protein h^–1^)**	**ACC (nmol α -ketobutyrate mg^–1^ h^–1^)**
	**A-tryptophan**	**P-tryptophan**		
AA1	14.97 ± 0.08^i−^^k^	69.20 ± 0.52^o^	22.18 ± 2.63^b−^^f^	−
AA2	15.37 ± 0.16^i−^^k^	59.92 ± 0.45^p^	20.58 ± 2.49^b−^^f^	164.89 ± 1.68^l^
AA3	13.25 ± 0.12^k−^^*m*^	69.78 ± 0.71^o^	20.55 ± 2.68^b−^^f^	−
AA7	24.23 ± 0.11^f^	195.60 ± 0.93^g^	24.82±2.81ba	209.75 ± 3.73^k^
AA10	44.88 ± 0.39^d^	903.31 ± 4.69^a^	22.41 ± 2.58^b−^^e^	700.30 ± 19.16^b^
AN3	15.93±0.30ji	772.99 ± 1.25^b^	23.75±2.88cb	−
AN4	21.35±1.53hg	41.35±0.19rq	11.44±1.46ji	−
AY3	64.36 ± 2.32^c^	635.93 ± 3.34^e^	19.63 ± 1.83^c−^^g^	473.43 ± 6.24^e^
AY5	13.38 ± 0.45^k−^^*m*^	731.55 ± 3.16^c^	22.28 ± 2.23^b−^^e^	254.96 ± 3.79^*j*^
BA18	12.44 ± 0.09^*m*^	693.51 ± 8.24^d^	23.21 ± 2.83^b−^^d^	157.80 ± 2.59^l^
BN4	16.45±1.37ji	149.88 ± 0.88^i^	23.52±2.19cb	282.01±2.10ji
BY1	13.38 ± 0.45^k−^^*m*^	75.96 ± 0.67^o^	24.73±2.64ba	207.25 ± 1.34^k^
BY4	29.59 ± 0.19^e^	570.47 ± 5.19^f^	28.97 ± 3.10^a^	468.77 ± 21.26^e^
BY5	14.21±0.12mj-	138.02 ± 0.98^*j*^	10.01±1.14ji	422.30 ± 9.95^f^
CA2	15.24 ± 0.15^i−^^k^	130.96±1.79kj	4.12±5.20lk	−
CA3	100.63 ± 0.98^a^	568.41 ± 6.62^f^	7.88±0.69kj	185.59±1.89lk
CA9	23.20±0.28gf	187.82 ± 1.87^g^	17.87±1.55gf	601.23 ± 11.62^c^
CA11	15.21 ± 0.41^i−^^k^	85.34 ± 1.08^*n*^	9.39±0.84ji	380.39 ± 7.88^g^
CN8	92.73 ± 1.71^b^	128.09 ± 0.74^k^	18.55 ± 1.77^e−^^g^	288.75 ± 9.10^i^
CN18	14.92 ± 0.21^i−^^l^	36.45 ± 0.34^r^	18.96 ± 1.73^d−^^g^	−
CN19	17.21 ± 0.15^i^	45.14 ± 0.23^*q*^	18.38 ± 1.82^e−^^g^	206.44 ± 5.25^k^
CoA3	12.56±0.32ml	73.31 ± 0.19^o^	4.96±0.25lk	−
CoA8	15.08 ± 0.19^i−^^k^	117.63 ± 0.39^l^	2.86 ± 0.32^l^	542.64 ± 9.33^d^
CoA9	20.28 ± 0.09^h^	41.35±0.19rq	22.03 ± 2.27^b−^^f^	950.40 ± 13.28^a^
CoA11	13.17 ± 0.13^k−^^*m*^	57.92 ± 0.29^p^	22.24 ± 2.29^b−^^e^	−
CoA12	12.43 ± 0.09^*m*^	96.16 ± 0.31^*m*^	12.26±1.25ih	−
CoN2	15.86±0.79ji	170.08 ± 1.27^h^	16.12±1.74hg	324.72 ± 1.19^h^

* A-(absence) of tryptophan; P-(presence) of tryptophan. Values (means of three repeats) not sharing a common letter differ significantly (P < 0.05) from each other.*

### Antifungal Activity

The interaction of selected rhizobacterial and two sugarcane pathogens, *S. scitamineum* and *C. paradoxa*, were studied here. Out of them, only 14 (52%) strains showed antifungal activity against *S. scitamineum*, and 20 (74%) strains were antagonistic to *C. paradoxa.* The isolates BY4 and AA7 exhibited antifungal activity against both pathogens (Table 1 and Supplementary Figure S2).

### 1-Aminocyclopropane-1-Carboxylic Deaminase Activity

All selected 27 strains were screened for ACCD activity, and 18 (67%) were able to grow on DF minimal salt medium 3–5 days after culture (Table 1). Based on these results, all 18 positive strains were further examined for the ACCD enzyme activity by quantifying the amount of α-ketobutyrate produced through the deamination of ACC. The higher level of ACCD activity was shown by the strain CoA9 (950.40 nmol α-ketobutyrate mg^–1^ h^–1^), followed by AA10, CA9, and CoA8 (700.3, 601.2, and 542.6 nmol α-ketobutyrate mg^–1^ protein h^–1^, respectively). Strain BA18 showed the lowest level of ACCD activity (157.80 nmol α-ketobutyrate mg^–1^ protein h^–1^) (Table 2).

### Nitrogenase Activity

All the selected strains exhibited nitrogenase activity, with varying levels for different strains (Table 2). Strain BY4 showed a relatively higher N-fixing activity in N-free culture medium, recording an acetylene reduction activity of 28.97 nmoL C_2_H_4_ mg ^–1^ protein h^–1^ followed by strains AA7 and BY1 (24.82 and 24.73 nmoL C_2_H_4_ mg^–1^protein h^–1^, respectively), under our experimental conditions. Other strains such as CoA8, CA2, CoA3, CA3, and CA11 produce much lower levels of acetylene.

### Gene Amplification of *nifH* and *acdS* Genes

**FIGURE 2:**
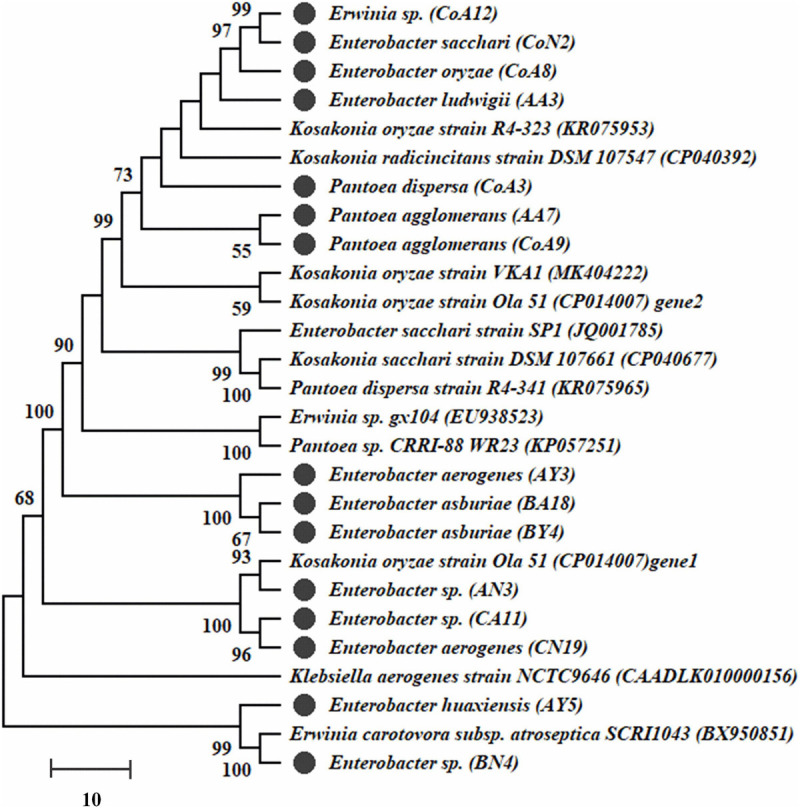
The dendrogram of *nifH* gene sequences of amplified fifteen strains was constructed by the neighbor-joining method. Bootstrap values of 1,000 replications are designated as% confidence values for certain branching.

Genomic DNA of all selected rhizobacteria was used to amplify *nifH* and *acdS* genes. Out of 27 rhizobacteria, 15 strains (CoA8, CoN2, AY3, AY5, AA3, AN3, BY4, BA18, BN4, CA11, CN19, CoA3, CoA9, CoA12, and AA7) had the *nifH* gene and a dendrogram was built (Figure 2). Those strains positive for *nifH* were sequenced. All sequences showed a 90–100% similarity level with the *nifH* gene, and they are submitted to the NCBI GenBank with accession numbers MT559588–MT559602. All 27 selected strains were also screened for *acdS* encoding ACCD enzyme. Only 12 strains showed amplification of the expected band size ∼750–755 bp of *acdS* gene (Supplementary Figure S3).

### Metabolic Characterization of Biolog Microarrays

**FIGURE 3:**
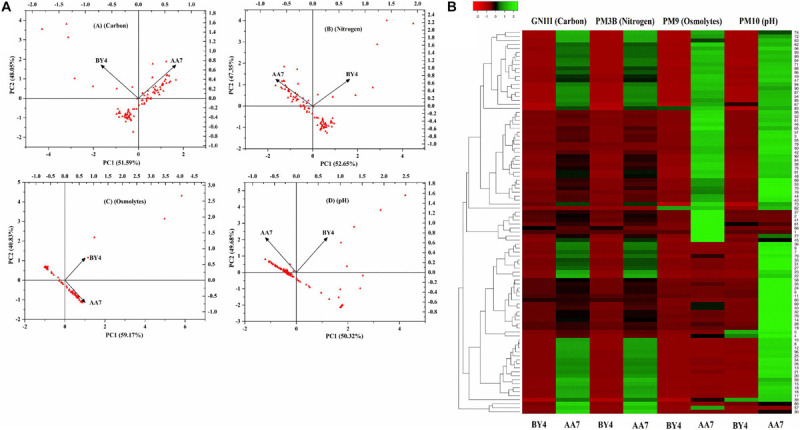
**(A)** Principal component analysis (PCA) of the most prominent rhizobacterial strains *P. dispersa* (AA7) and *E. asburiae* (BY4) were examined for different carbon, nitrogen, osmolytes, and pH in relation with diverse media compositions as well as stress responses based on BIOLOG^(R)^ microplates. **(B)** Heat map graph showing the growth of BY4 and AA7 strains as the colored graphical form in every 96 different substrates of GNIII, PM3B PM9, and PM10 Biolog plates.

Out of 27 strains, only two of them (BY4 and AA7) were selected for further studies to understand their genotypic differences, stress response, growth, and survival on diverse media, which is an essential criterion for their application in the field. A PCA scatter plot of Biolog data showed that carbon components explained 51.59% (PC1) and 48.05% (PC2) while N components accounted for 52.65% (PC1) and 47.35% (PC2) of the metabolic variation between isolates, whereas osmolyte scatter plots accounted for 59.17% (PC1) and 40.83% (PC2) of the variance. For pH, PC1 and PC2 explained 50.32 and 49.68%, respectively, of the total variance (Figure 3A). Based on the PCA scores for different substrates, the diversity index parameters were measured (Supplementary Table S6). The heatmap graph representing the growth shown by BY4 and AA7 strains in 96 different substrates of GNIII, PM3B, PM9, and PM10 Biolog plates is presented in colored graphical form. The graph showed that the relative abundance based on different substrate utilizations in each well (total of 96 wells) and clustering specifies the similarity of all 96 different substrates between the selected strains. Both strains confirmed the utilization of various carbon and nitrogen substrates with considerable osmotic and pH stress tolerance, and AA7 showed better results as compared to BY4 (Figure 3B).

### Colonization Pattern of *E. asburiae* and *P. dispersa* on Sugarcane

**FIGURE 4:**
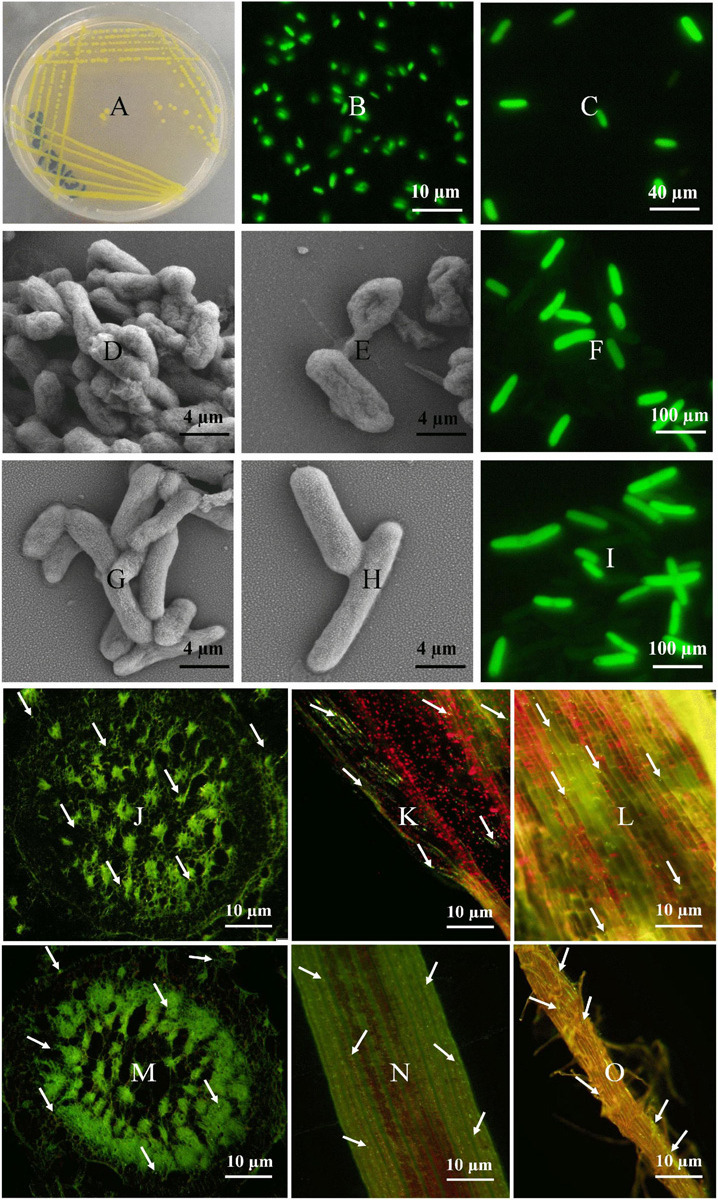
Fluorescence micrographs of GFP-tagged *P. dispersa* (AA7) and *E. asburiae* (BY4) rhizobacteria: **(A–C)** is a plasmid pPROBE-pTetr-OT containing the green fluorescent protein (GFP). **(D,E)** and **(G,H)** is the colony morphology of *P. dispersa* (AA7) and *E. asburiae* (BY4) observed by SEM. **(F,I)** are the GFP/pPROBEpTet^r^-TT tagged *P. dispersa* and *E. asburiae*. **(J–L)** and **(M–O)** CLSM of GFP/pPROBEpTet^r^-TT-tagged *P. dispersa* and *E. asburiae* colonizing root, leaf, and stem tissues of micropropagated sugarcane plantlets. The images represent bacterial cells in green dots (white arrow), indicating the colonization of rhizobacteria with autofluorescence in every part of sugarcane tissues, respectively.

Colonization of *E. asburiae* (BY4) and *P. dispersa* (AA7) in sugarcane was investigated through their tissue localization by CLSM. Both strains fix N, showed different PGP traits and protection against sugarcane pathogens (*S. scitamineum* and *C. paradoxa*), and survived under different stress (NaCl salinity and pH extremes) conditions. Plasmid pPROBE-pTetr-OT containing the green fluorescent protein (GFP) (Figures 4A–C). The GFP-tagged strains inoculated in 60 day-old sugarcane plants were monitored 3 days post-inoculation, and both strains were found in sugarcane inside the root, leaf, and stem tissues (Figures 4J–O). After inoculation, bacterial cells were mostly dispersed on the elongation and differentiation zones of the main roots and lateral root junctions. Root hairs had the greatest bacterial population density. Microscopic images showed the rod-shaped morphology of both selected strains (Figures 4D–I). Both strains were gram-negative, non-spore-forming bacteria with yellow color and smooth colonies.

### Physiological Parameters and Activity of Defense-Related Enzymes in Sugarcane

All measured physiological parameters i.e., plant height, shoot and root weight, leaf area, chlorophyll content, and photosynthesis, of GT11 and GXB9 plants inoculated with rhizobacterial strains (AA7 and BY4) was favorably influenced by the bacteria and showed significant differences compared to the control up to 90 DAI (Table 3). Strain BY4 showed a considerable increase in all growth parameters at 30 and 60 DAI, whereas at 90 days AA7 was more efficient than BY4 under greenhouse conditions. Strain AA7 showed a maximum increase in plant height (54 and 52%), root weight (57 and 53%) chlorophyll content (24 and 33%), and photosynthesis (100 and 133%) in GT11 and GXB9 varieties as compared to control at 90 DAI. Strain BY4 had greater effects on shoot weight and leaf area than AA7 in GT11, with 86 and 106% increase for shoot weight and leaf area. In GXB9, the highest 57% increase of shoot weight and 64% of leaf area was observed with the inoculation of AA7 as compared to control (Table 3 and Supplementary Figure S4).

**TABLE 3:** The effect of *P. dispersa* (AA7) and *E. asburiae* (BY4) rhizobacterial inoculum on the physiological parameters of sugarcane varieties GT11 and GXB9 at different time intervals in the greenhouse experiment.

**Parameters**	**Treatments**	**30 Days**		**60 Days**		**90 days**		**Percentage change over control at 90 days**	
		**GT11**	**GXB9**	**GT11**	**GXB9**	**GT11**	**GXB9**	**GT11**	**GXB9**
Plant height (cm)	Control	25.22 ± 0.38^c^	26.65 ± 0.40^c^	37.32 ± 0.56^b^	42.40 ± 0.64^b^	56.81 ± 0.85^c^	61.32 ± 0.92^c^	**−**	**−**
	BY4	41.29 ± 0.62^a^	40.29 ± 0.60^a^	56.85 ± 0.85^a^	56.85 ± 0.85^a^	75.91 ± 1.14^b^	87.60 ± 1.31^b^	34	43
	AA7	36.43 ± 0.55^b^	37.43 ± 0.56^b^	55.49 ± 0.83^a^	58.50 ± 0.88^a^	87.60 ± 1.31^a^	92.97 ± 1.40^a^	54	52
Shoot weight (g)	Control	10.44 ± 0.16^c^	10.79 ± 0.16^c^	31.75 ± 0.48^c^	37.99 ± 0.57^c^	66.14 ± 0.99^c^	80.04 ± 1.20^c^	**−**	**−**
	BY4	20.52 ± 0.31^a^	19.97 ± 0.30^a^	73.40 ± 1.10^a^	62.72 ± 0.94^a^	122.72 ± 1.84^a^	117.86 ± 1.77^b^	86	47
	AA7	14.75 ± 0.22^b^	18.61 ± 0.28^b^	57.70 ± 0.87^b^	59.66 ± 0.90^b^	111.13 ± 1.67^b^	125.33 ± 1.88^a^	68	57
Root weight (g)	Control	1.21 ± 0.02^c^	1.85 ± 0.03^c^	2.29 ± 0.03^c^	3.40 ± 0.05^c^	9.57 ± 0.14^c^	11.32 ± 0.17^b^	**−**	**−**
	BY4	4.67 ± 0.07^a^	4.97 ± 0.07^a^	5.97 ± 0.09^a^	6.87 ± 0.10^a^	14.30 ± 0.21^b^	16.76 ± 0.25^a^	49	48
	AA7	3.21 ± 0.05^b^	4.31 ± 0.06^b^	5.42 ± 0.08^b^	6.52 ± 0.10^b^	15.05 ± 0.23^a^	17.31 ± 0.26^a^	57	53
Leaf area (cm^2^)	Control	30.78 ± 0.46^c^	31.38 ± 0.47^c^	168.82 ± 2.53^c^	177.24 ± 2.66^c^	641.83 ± 9.63^c^	788.58 ± 11.84^c^	**−**	**−**
	BY4	40.48 ± 0.61^a^	34.84 ± 0.52^b^	303.82 ± 4.56^a^	312.65 ± 4.96^a^	1319.71 ± 19.81^a^	1222.88 ± 18.35^b^	106	55
	AA7	33.45 ± 0.50^b^	45.35 ± 0.68^a^	261.05 ± 3.92^b^	263.41 ± 3.95^b^	1129.11 ± 16.95^b^	1295.72 ± 19.45^a^	76	64
Chlorophyll content (SPAD units)	Control	15.43 ± 0.23^b^	16.39 ± 0.25^c^	31.62 ± 0.47^b^	33.81 ± 0.51^c^	36.55 ± 0.55^b^	32.97 ± 0.49^b^	**−**	**−**
	BY4	19.41 ± 0.29^a^	20.80 ± 0.31^a^	36.36 ± 0.53^a^	37.60 ± 0.56^b^	43.48 ± 0.65^a^	42.35 ± 0.64^a^	19	28
	AA7	18.83 ± 0.28^a^	19.49 ± 0.29^b^	35.59 ± 0.55^a^	40.44 ± 0.61^a^	45.29 ± 0.68^a^	43.78 ± 0.66^a^	24	33
Photosynthesis (μ mol CO_2_ m^–2^ s^–1^)	Control	5.68 ± 0.09^c^	6.75 ± 0.10^c^	8.38 ± 0.13^c^	9.76 ± 0.15^b^	11.23 ± 0.17^c^	12.36 ± 0.19^c^	**−**	**−**
	BY4	9.71 ± 0.15^a^	10.78 ± 0.16^a^	16.92 ± 0.25^a^	18.27 ± 0.27^a^	19.50 ± 0.29^b^	27.38 ± 0.43^b^	74	122
	AA7	9.01 ± 0.14^b^	9.80 ± 0.15^b^	15.53 ± 0.23^b^	18.70 ± 0.28^a^	22.47 ± 0.37^a^	28.83 ± 0.41^a^	100	133

*Means followed by the same letter are not significantly different (P ≤ 0.05).*

**FIGURE 5:**
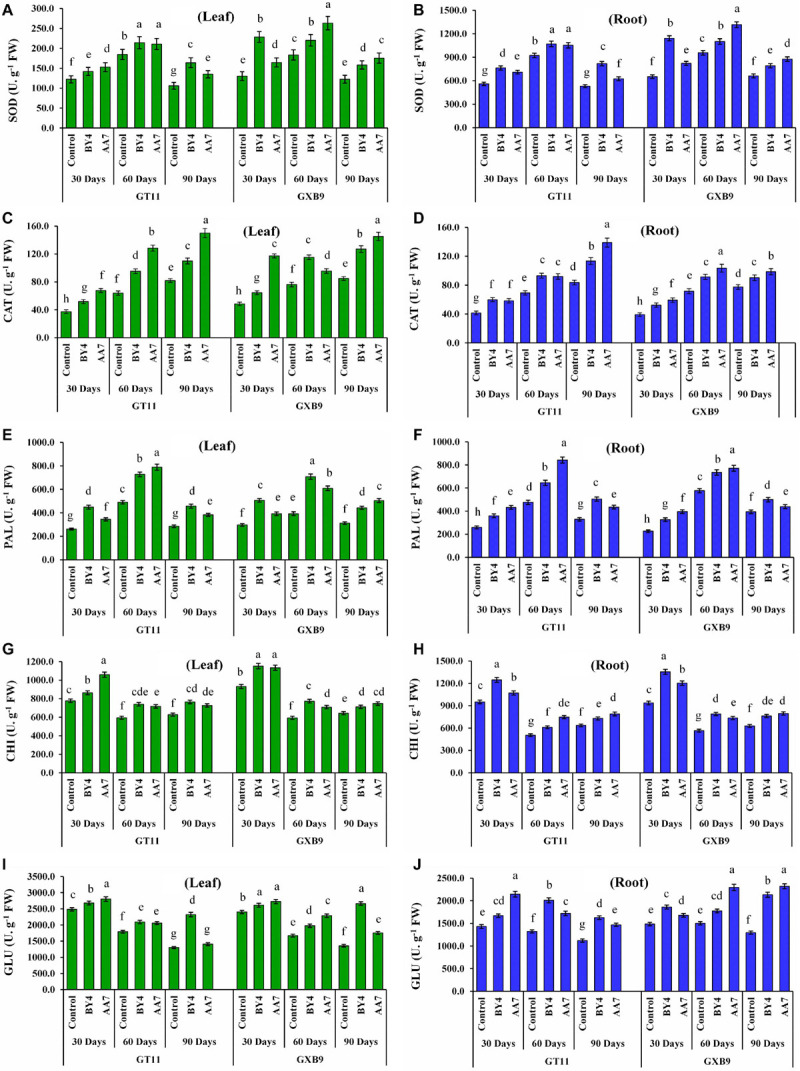
The effect of nitrogen-fixing rhizobacterial strains *P. dispersa* (AA7) and *E. asburiae* (BY4) inoculation on enzyme activities: **(A,B)** superoxide dismutase (SOD), **(C,D)** catalase (CAT), **(E,F)** phenylalanine ammonia-lyase (PAL), **(G,H)** chitinase (CHI), and **(I,J)** β-1,3-glucanase (GLU) in leaf and root tissues of sugarcane varieties (GT11 and GXB9). Sugarcane leaf and root tissues were harvested at 30, 60, and 90 DAI. Different letters show significant differences between treatments at *p* < 0.05.

Inoculation of BY4 and AA7 strains enhanced the activity of SOD, CAT, PAL, CHI, and GLU enzymes in leaf and root tissues of both sugarcane varieties (GT11 and GXB9) compared to control (Figure 5). Superoxide dismutase activity in roots was remarkably higher than that of leaf irrespective of the treatment. Similarly, in all treatments SOD activity in leaf and root tissues of both sugarcane varieties showed an increasing trend till 60 DAI and then declined (Figures 5A,B). There was no discernable pattern or difference in SOD activity between AA7- and BY4-inoculated plants except for a relatively 43% higher activity observed in leaf tissues of AA7-inoculated GXB9 on 60 DAI and 75% in BY4-inoculated GXB9 on 30 DAI as compared to control. Inoculation of GT11 and GXB9 with AA7 maximum increased CAT activity in leaf (83%) and roots (66%) remarkably compared with control at 90 days. In general, AA7 was found to be more effective than BY4 in inducing CAT activity in both tissues and varieties (Figures 5C,D). Unlike SOD and PAL, the activity of CAT in leaf and roots in both varieties gradually increased as plant growth advanced till 90 DAI, and this effect was treatment-independent (Figures 5C,D). The overall activity of PAL also showed a pattern somewhat similar to that of SOD with the activity peaking on 60 DAI in all treatments (Figures 5E,F). However, unlike SOD, the difference in PAL activity between leaf and root tissues was negligible in most treatments till 90 DAI (Figures 5E,F). Both AA7 and BY4 did not differ significantly in eliciting PAL activity in leaf and roots in both varieties in all but for 60 DAI in GT11. Maximum CHI activity was recorded in all analyzed samples on 30 DAI with 45% in root tissues of GXB9 inoculated with BY4 (Figures 5G,H). In general, CHI activity was more or less similar in both leaf and roots irrespective of variety and treatments, but the bacterial inoculation caused a significant increase in activity compared to control. Both bacteria showed a similar capacity for CHI induction in leaf and roots in both varieties. Like all other enzymes studied, GLU activity was maximum of 13% increased by AA7 inoculation in leaf tissues in GT11 and GXB9, and in all the measured time points, but it did not change greatly with time till 90 DAI (Figures 5I,J). There was no consistent difference in enzyme induction between AA7 and BY4 strains in both varieties.

### Gene Expression for *nifH* and Pathogen Defense-Related Genes

**FIGURE 6:**
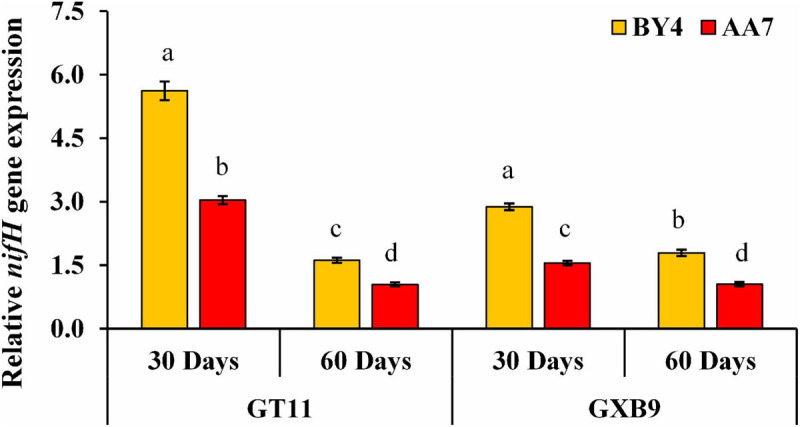
Expression analysis of *nifH* gene after rhizobacterial inoculation of *P. dispersa* (AA7) and *E. asburiae* (BY4) by qRT-PCR in leaf tissues of GT11 and GXB9 sugarcane varieties harvested at 30 and 60 DAI. All data points were the means ± SE (*n* = 3) and standardized to the *GAPDH* expression level. Different lowercase letters display a significant difference at *p* < 0.05.

The *nifH* gene expression was quantified using qRT-PCR. The gene expression was higher in AA7- and BY4-inoculated plants of both varieties on 30 DAI compared with that of 60DAI, with the highest expression recorded in BY4*-*inoculated GT11 (Figure 6 and Supplementary Table S7).

**FIGURE 7:**
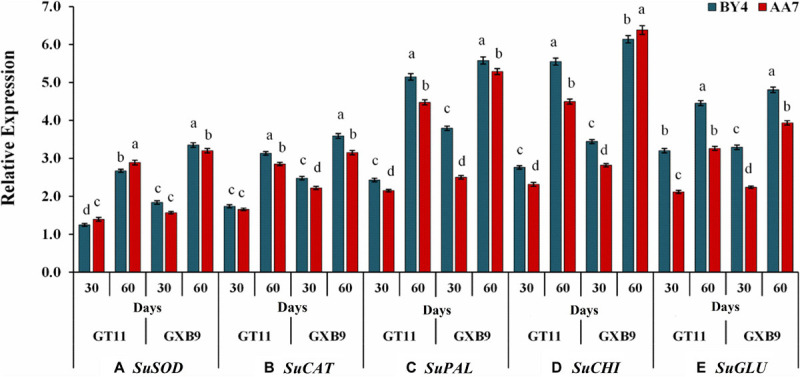
The effect of rhizobacteria *P. dispersa* (AA7) and *E. asburiae* (BY4) observed on the relative gene expression levels of **(A)** superoxide dismutase (SOD), **(B)** catalase (CAT), **(C)** phenylalanine ammonia-lyase (PAL), **(D)** chitinase (CHI), and **(E)** β-1,3-glucanase (GLU) in two different sugarcane varieties. Plant leaf samples were harvested after 30 and 60 days of treatment. All data were standardized to the GAPDH expression level and expressed as the mean ± SE (*n* = 3). The different letters on the error bars show significant differences, whereas the same letters above the bars indicate no difference among treatments at *p* < 0.05.

The expression of *SuSOD* increased with the time of the experiment with GXB9 showing a slightly but significantly higher expression than that of GT11 till 60 DAI. There was no significant difference between AA7 and BY4 for inducing *SuSOD* gene expression in both varieties (Figure 7). More or less a similar pattern was evident for expression of *SuCAT*, *SuPAL*, *SuCHI*, and *SuGLU* genes on 30 and 60 DAI in both varieties, though the relative expression was much higher for *SuPAL* and *SuCHI* than the other two genes (Figure 7 and Supplementary Table S8).

### Quantification of BNF Using ^15^N and Total N Content in Sugarcane Varieties

**FIGURE 8:**
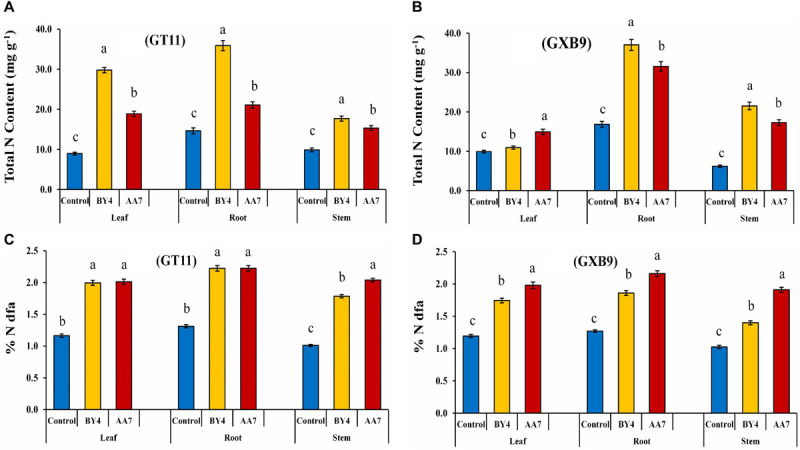
Estimation of BNF by *P. dispersa* (AA7) and *E. asburiae* (BY4) rhizobacteria in sugarcane plant tissues for dry biomass (root, stem, and leaf) of GT11 and GXB9 varieties grown in pots containing ^15^N-labeled soil (values represent mean of three replicates). **(A,B)** Total N content and **(C,D)** % N dfa. Different letters indicate significant differences among treatments at *p* < 0.05.

The BNF in GT11 and GXB9 varieties following their inoculation with rhizobacteria was measured by the ^15^N isotope dilution technique at 180 DAI. The results showed that BY4 and AA7 inoculation increased the N content of leaf, root, and stem of both varieties (Figure 8 and Supplementary Table S9). The BNF contribution varied between the tested sugarcane varieties. The highest BNF contribution was found in the root of both varieties with the inoculation of BY4 (36–37% more than the control), while the inoculation of AA7 resulted in total N measuring 21–32% more than that of control (Figures 8A,B). The leaves also showed a significant increase in total N content in GT11 with BY4, but in GXB9, the stem of BY4-inoculated plants showed the maximum N content compared to control. The roots of both varieties showed significantly higher N compared with control, irrespective of the bacterial strain used for inoculation. Both strains had a higher impact on N content in both sugarcane varieties as compared to control (Figures 8C,D).

## Discussion

In this study, we mainly focused on Enterobacter, Pantoea, and Erwinia genera isolated from the sugarcane rhizosphere. A total of 27 strains were identified based on their nitrogen-fixing, PGP, and antifungal activities against sugarcane fungal pathogens and established their taxonomic identity through 16S rRNA gene sequencing. These strains are classified as *E. oryzae* (CoA8), *E. sacchari* (CoN2), *E. aerogenes* (AY3 and CA11), *E. huaxiensis* (AY5), *E. ludwigii* (AA3 and AN4), *E. cloacae* (AA10 and CA9), *E. asburiae* (BY4, BA18, and CN18), *E. tabaci* (CA2), *E. cancerogenus* (CA3 and CN19), *E. mori* (CN8), *E.* species (AN3 and BN4), *P*. *dispersa* (CoA3, AA7, and BY5), *P. agglomerans* (CoA9 and AA2), *P*. species (CoA11 and AA1), and *Erwinia* sp. (CoA12 and BY1). All selected strains are gram-negative, rod-shaped, and non-spore-forming bacteria. Among these, *E. asburiae* and *P*. *dispersa* were the most dominant species. Only a few reports are available on the isolation of diazotrophic endophytic strains i.e., *Pantoea* sp. (Loiret et al., 2004), *P. agglomerans* (Quecine et al., 2012), *P. dispersa* (da Silveira et al., 2018), *Enterobacter* sp. (Zhu et al., 2012), *E. sacchari* (Zhu et al., 2013), and *E. roggenkampii* (Guo et al., 2020) from sugarcane. However, no report on the isolation of diazotrophic *P. dispersa* and *E. asburiae* associated with the sugarcane rhizosphere.

Two strains, *E. asburiae* (BY4) and *P. dispersa* (AA7), displayed all PGP traits and prompting to study of its PGPR potential for sugarcane. Several *Enterobacter* sp. and *Pantoea* sp. have been reported as phosphate solubilizers (Mishra et al., 2011; Dhole et al., 2016; Sarkar et al., 2018; Shabanamol et al., 2018; Macedo-Raygoza et al., 2019). Phosphate-solubilizing microorganisms enhance phosphorus accessibility to plant by altering inorganic phosphorus into soluble phosphorus form (Dastager et al., 2009; Wang et al., 2009) and greatly improve plant growth, particularly root development. In addition to phosphate solubilization, siderophore production is another essential PGP trait, facilitating plant-available iron (Fe^3+^) in the rhizosphere (Beattie, 2006). The ability of rhizobacteria to promote root growth via IAA production will have an additive effect on boosting plant nutritional enhancement by PGPR. Auxins produced by rhizospheric bacteria had a major effect on cellular division, and more specifically on root development (Cherif-Silini et al., 2016). In previous studies, many strains have been reported as IAA producers, which may explain their ability to promote growth in sugarcane (Li et al., 2017; Rampazzo et al., 2018; Guo et al., 2020; Singh et al., 2020). Also, several PGP strains from *Pantoea* sp. were reported to produce phytohormone production (Dastager et al., 2009; Bible et al., 2016). In our study, all bacterial strains produced a significant amount of IAA ranging from 36.45 to 903.31 μg mL^–1^, with the AA10 strain being the greatest producer.

Ethylene is a phytohormone that controls plant growth and stress responses (Bleecker and Kende, 2000). One of the principal mechanisms by which DPGPRs exert their beneficial effects on plants under stress is possibly through ACCD activity. ACCD-producing bacteria convert the ethylene precursor ACC into α-ketobutyrate and ammonia, thus reducing the endogenous level of stress hormone ethylene which modulates plant tolerance to environmental stresses and improves plant growth (Glick et al., 1998; Poel and Straeten, 2014). ACCD activity has been recognized as a biomarker for plant growth-promoting bacteria (PGPB) (Glick et al., 2007). Bacteria producing ACCD are generally isolated on minimal media containing ACC as the sole source of nitrogen, and ACCD activity was identified by bacteria that are grown on the ACC media (Penrose and Glick, 2003). Earlier studies also reported that some bacterial strains that grew on the ACC medium did not express ACCD activity because strains without ACCD can also be grown on the ACC medium (Li et al., 2011). The presence of trace nitrogen in medium and agar components possibly supports the growth of some bacteria without ACCD activity, whereas a few bacteria with ACCD may not confirm ACCD activity in free-living states (Blaha et al., 2006; Nascimento et al., 2012), and a pyridoxal 5′-phosphate (PLP)-dependent deaminase that is not an ACCD may express less non-specific ACCD activity (McDonnell et al., 2009; Nascimento et al., 2014). Nascimento et al. (2014) described ACCD activity in many *Bacillus* strains but did not identify the *acdS* gene. Therefore, unambiguous detection of the ACC deaminase structure gene (*acdS*) is essential to confirming bacteria producing ACCD activity (Onofre-Lemus et al., 2009). In this study, 18 strains produced ACCD activity and positive *acdS* gene amplification was confirmed only for 12 strains, possibly for the above reasons. In previous reports, *Pantoea* and *Enterobacter* strains isolated from other crops were confirmed to have ACCD activity (Canamas et al., 2009; Sarkar et al., 2018; Nascimento et al., 2020), and the *Enterobacter* sp. EN-21 strain producing ACCD promoted sugarcane growth under salt-stress conditions (Kruasuwan and Thamchaipenet, 2018).

The sugarcane crop is normally affected by many fungal pathogens. Sugarcane smut and pineapple disease caused by *S. scitamineum* and *C. paradoxa*, respectively, are very important fungal diseases, and they caused numerous commercial varieties out of production (Que et al., 2012). Sugarcane is a perennial crop that harbors many useful bacteria in its rhizosphere, and the use of growth-promoting bacteria identified from rhizosphere assemblage is becoming a practically useful approach to improve crop nutrition and biocontrol of microbial pathogens (Singh et al., 2020). In the current paper, 20 strains showed the antagonistic potential to *C. paradoxa*, whereas 14 strains restricted *S. scitamineum* growth with two strains, BY4 and AA7, which proved to very promising to control fungal pathogens. Secondary metabolites such as HCN and ammonia are produced by several bacterial strains, and they play important roles in controlling fungal diseases in several plants (Blumera and Haas, 2000; Martínez-Viveros et al., 2010; Olanrewaju et al., 2017). In this study, HCN and ammonia production by DPGPR BY4 and AA7 was observed, proving that these strains are valuable for controlling diseases such as smut and pineapple disease. Whereas some PGPR-producing HCN failed to show biocontrol activity in response to fungal pathogens but improved nutrient accessibility in plants (Blom et al., 2011; Rijavec and Lapanje, 2016).

The N-fixing activity of all the selected bacterial strains was determined with the ARA method, and BY4 showed a higher nitrogenase activity than AA7 in an N-free medium. Screening for N-fixing genes (*nifH*) in the selected strains by PCR was found to have just 15 strains with the *nifH* gene among all selected strains. Amplification of the *nifH* gene established the potential nitrogen-fixation capacity of strains (Zehr and Capone, 1996). In contrast, the lack of *nifH* gene amplification does not necessarily imply that the strains are not capable of BNF as the *nifH* gene could display different nucleotide sequences among as well as within species (Zehr et al., 2003). Based on all PGP traits and nitrogen-fixing ability, we selected *P. dispersa*-AA7 and *E. asburiae*-BY4 for additional studies. We quantified the expression of the *nifH* gene, and less increase in *nifH* expression was observed at 60 DAI of BY4 and AA7 strains as compared to day 30 in both sugarcane varieties. The reason might be that these strains were not colonized properly at that time, due to some environmental conditions or that the sampling time was not suitable for these sugarcane varieties. The expression of the *nifH* gene indicated the BNF activity of these nitrogen-fixing bacteria. Singh et al. (2020) reported the expression of the *nifH* gene in sugarcane plants inoculated with *B. megaterium* and *B. mycoides* PGP strains. ^15^N isotope dilution and N balance assessments with different sugarcane varieties identify their capacity to acquire atmospheric N through BNF microorganisms (Urquiaga et al., 1992). The results obtained in this study with the ^15^N isotope dilution method proved significant BNF by the selected strains and their contribution to the sugarcane N requirement.

A complex relationship between soil type and plant species influences the bacterial population and composition in the rhizosphere (Marschner et al., 2001). Compatibility between the composition of the host plant root exudate and the potential of the PGPR to use these compounds is one significant factor for PGPR existence (Strigul and Kravchenko, 2006). Phenotypic profiling is significant for discerning genotypic differences, stress responses, and growth media composition, and it varies with environmental conditions (Chojniak et al., 2015). Compared to other standard bacterial cultivation approaches, various substrate consumption trends by PGPR using the Biolog plate provide a comparatively quicker and more sensitive way of evaluating improvements in microbial functional diversity (Nautiyal, 2009). This approach is gaining momentum due to its simplicity and pace of success (Winding and Hendriksen, 2007; Mishra and Nautiyal, 2009; Nautiyal, 2009). An organism’s metabolic properties can contribute to a specific niche adaptation. Metabolic variations have made it possible for DPGPR to adapt to particular conditions, such as soil and plant tissues. The BIOLOG metabolic profiling study is a valuable tool to characterize the microbial populations for substrate utilization, and pH and stress tolerance. The observed phenotypes suggest that both BY4 and AA7 strains can utilize various metabolic substrates. In previous reports, it was also recommended that strains with broad metabolite tolerance are more suitable for plant nodulation and plant growth (Wielbo et al., 2007; Mazur et al., 2013). However, bacterial growth on microtiter plates often depends on multiple aspects of its culture, such as microtiter plate, replication, conditions of incubation, and plate monitoring (Preston-Mafham et al., 2002). Competitive rhizosphere colonization is part of PGPR–plant interactions (Chin-A-Woeng et al., 2000; Timmusk et al., 2005). Therefore, to develop the PGPR as a beneficial bioinoculant application, large-scale multilocation field trials are needed. We also observed that sugarcane plants inoculated with BY4 and AA7 showed a considerable increase in plant height, shoot and root weight, leaf area, chlorophyll content, and photosynthesis as compared to control. Strain BY4 showed a considerable increase in all growth parameters at 30 and 60 DAI, whereas at 90 days AA7 was more efficient than BY4 under greenhouse conditions. The reason for this could be that strain BY4 may be a good colonizer during the initial stages but later did not proliferate properly in the plants due to various environmental or plant host-dependent reasons, whereas strain AA7 grew effectively after inoculation, and promoted plant growth.

Understanding the molecular basis of sugarcane–rhizobacteria interactions may lead to practically useful technological solutions for improving sugarcane growth and yield. We observed the colonization of GFP-tagged *E. asburiae* and *P. dispersa* strains in sugarcane plantlets. When inoculated individually, both strains showed colonization in the entire plant body, and *E. asburiae* showed better colonization than *P. dispersa* in sugarcane. Many researchers have implemented *in situ* visualizations of bacterial cells in the rhizosphere and on root surfaces using GFP as a marker to analyze plant–microbe interactions. The capability of these strains to colonize sugarcane plants and function as an effective plant growth-promoting bacteria (PGPB) is important as observed for *E. roggenkampii*, *Microbacterium* sp., *Bacillus*, and *Pseudomonas* strains previously (Lin et al., 2012; Li et al., 2017; Guo et al., 2020; Singh et al., 2020). To the best of our knowledge, this is the first report that elucidates the interaction and colonization process of *E. asburiae* and *P. dispersa* strains in sugarcane.

Reactive oxygen species are a common plant metabolite produced under normal growth and biotic and abiotic stress conditions, and it is a temporary plant defense mechanism during plant–microbe interactions (Yan et al., 2007; Wasim et al., 2009; Nanda et al., 2010). ROS is continuously being produced at basal levels under favorable conditions and scavenged by various antioxidant enzymes; they are unable to inflict harm (Foyer and Noctor, 2005). In plants, ROS help to withstand even symbiotic bacteria until plants benefit from symbiosis (Santos et al., 2001). Enzymes such as SOD and CAT are involved in ROS metabolism and plant cell defense process. The expression of SOD and CAT is induced by environmental stress stimuli and pathogens (Dat et al., 2000; Chen et al., 2012). PAL provides the plant’s physiological and systemic support by changing L-phenylalanine to trans-cinnamic acid and ammonia. In rice, microbial treatment has been shown to increase the activity of PAL and the aggregation of polyphenols in the leaves, thus helping to enhance stress conditions (Bhattacharyya et al., 2020). By the production of defense-related antioxidant enzymes and molecules, PGPR primes host plants to avoid pathogen attacks (Choudhary et al., 2007). The biocontrol mechanisms consist of the production of cell wall degrading enzyme production and the activation of systemic resistance defense processes (Hayat et al., 2012). Plant chitinases and β-1,3-glucanase have recognized PR proteins, widely dispersed in higher plants, contributing to plant defense mechanisms in response to numerous pathogens, as well as some abiotic stresses (Viswanathan and Samiyappan, 2001; Lawton et al., 2005; Wan et al., 2011). In such a situation, the implementation of PGPR could come to a great rescue. Increased activity of defense-related enzymes in diazotrophic PGPR-treated sugarcane plants eventually led us to conclude that both strains can promote induced systemic resistance, a condition of increased defensive ability in sugarcane plants. In this study, we also quantified the activity and expression of five different defense-related enzymes (SOD, CAT, PAL, CHI, and GLU), and the pattern observed for enzyme activity and expression was dissimilar at different time intervals. As observed previously in other systems (Ara et al., 2013; Du et al., 2013; Su et al., 2013, 2014). This is because gene expression is controlled at many different stages and in many different ways such as transcriptional and posttranscriptional regulations (RNA processing, RNA stability, etc.). Previous reports show that rhizosphere microbial inoculation enhances growth and antioxidant enzyme activities in different plants as compared to control under normal conditions (Bhattacharyya et al., 2020; Wang et al., 2020). Increased expression of *ScChi*, *ScGluD1*, *SuCAT*, *SuSOD*, and S*uPAL* genes was observed following artificial inoculation of PGP strains in GT11 and GXB9 (Singh et al., 2020) and smut pathogen in sugarcane varieties (Su et al., 2013, 2014; Singh et al., 2018, 2019).

## Conclusion

The present study shows the existence of a genetically diverse microbiome population of diazotrophic plant growth promoting rhizobacteria of *Enterobacter* and *Pantoea* genera in the sugarcane rhizosphere. All isolated rhizobacteria elicited different PGP traits, N fixation, and biocontrol potential in response to sugarcane pathogens. The application of efficient N-fixing PGPR has the potential to increase crop production. To the best of our knowledge, this is the first report of growth promotion, N-fixation, and the expression of defense-related genes in sugarcane plants by rhizobacteria *E. asburiae* and *P. dispersa* under greenhouse conditions. Therefore, both strains may be used to study plant–microbe interactions that enhance sugarcane productivity, soil fertility, protection against different biotic stresses, and environmental sustainability.

## Data Availability Statement

The datasets presented in this study can be found in online repositories. The names of the repository/repositories and accession number(s) can be found in the article/ Supplementary Material.

## Author Contributions

PS, RS, L-TY, and Y-RL: planning the proposal and experiments. PS, RS, and H-BL: completing the experiments. AS and D-JG: data analysis. MS, MM, X-PS, and KV: validation. L-TY and Y-RL: investigation and resources. RS and PS: writing of the original draft. PL, L-TY, and Y-RL: review and editing. YL: supervision and project administration. All authors contributed to the article and approved the submitted version.

## Conflict of Interest

The authors declare that the research was conducted in the absence of any commercial or financial relationships that could be construed as a potential conflict of interest.
