# Supplementary material for: Diazotrophic Bacteria Pantoea dispersa and Enterobacter asburiae Promote Sugarcane Growth by Inducing Nitrogen Uptake and Defense-Related Gene Expression
Source: Front Microbiol. 2021 Jan 12;11:600417. doi: 10.3389/fmicb.2020.600417 (PMC7835727; doi:10.3389/fmicb.2020.600417)
Supplement: Supplementary file 1 [file Data_Sheet_1.docx]

Supplementary Material

**TABLE S1** List of different medium composition used in this research work for the isolation and screening of DPGPR strains from sugarcane.

| **S. No.** | **Medium name and composition (g L^-1^)** |
| --- | --- |
| Ashbey’s Medium | Mannitol; 15, CaCl_2_.2H_2_O; 0.2, MgSO_4_.7H_2_O; 0.2, MoO_3_ (10% solution); 0.1 mL, FeCl_3_ (10% solution); 0.05 mL, Agar; 15 |
| Yeast Mannitol Agar Medium | Mannitol; 15, K_2_HPO_4_; 0.5, Yeast Extract; 0.4, MgSO_4_.7H_2_O; 0.2, NaCl; 0.1, Agar; 15 |
| JNFb medium (Baldani et al. 1992) | Malic acid, 5.0; K_2_HPO_4_, 0.6; KH_2_PO_4_, 1.8; MgSO_4_.7H_2_O, 0.2; NaCl, 0.1; CaCl_2_.2H_2_O, 0.02.  Micronutrient solution: (CuSO_4_.5H_2_O, 0.04; ZnSO_4_.7H_2_O, 0.12; H_3_BO_3_, 1.40; Na_2_MoO_4_.2H_2_O, 1.0; MnSO_4_. H_2_O, 1.175) (g L^-1^).  2 mL; bromothymol blue (5 g L^−1^ in 0.2 N KOH), 2 mL; Fe EDTA (16.4 g L^−1^), 4 mL; vitamin solution (biotin, 10 mg; pyridoxal-HCl, 20 mg, dissolved in a hot-water bath. Complete to 100 mL by adding distilled water), 1 mL; KOH, 4.5g. Add distilled water to bring the total solution to 1,000 mL. Adjust the pH to 6.8 with KOH. |
| LGI Medium | Sucrose; 5, KH_2_PO_4_; 0.6; K_2_HPO_4_; 0.2; MgSO_4_.7H_2_O; 0.2, CaCl_2_.2H_2_O; 0.02, FeCl_3_; 1, Na_2_MoO_4_. 2H_2_O; 2 mg, Bromothymol Blue Solution; 5 mL, Agar; 1.75 |
| Nutrient Agar | Peptone; 5, NaCl; 5, Yeast Extract; 2, Beef Extract; 1, Agar; 15 |
| Potato Dextrose Agar | Potatoes (infusion from); 200, Dextrose; 20, Agar; 15 |
| DF salts minimal medium | Glucose; 2, Gluconic acid; 2, Citric acid; 2, KH_2_PO_4_; 4, Na_2_HPO_4_; 6, MgSO_4_**.**7H_2_O; 0.2, Micro nutrient solution (in mg) (CaCl_2_; 200, FeSO_4_.7H_2_O; 200, H_3_BO_3_; 15, ZnSO_4_.7H_2_O; 20, Na_2_MoO_4_; 10, KI; 10, NaBr; 10, MnCl_2_; 10, COCl_2_; 5, CuCl_2_; 5, AlCl_3_; 2, NiSO_4_; 2. |
| Luria Bertani Broth | Casein enzymic hydrolysate; 10, Yeast extract; 5, Sodium chloride; 5. |

**TABLE S2** Primers used for rhizobacterial identification, *nifH,* and *acdS* gene amplification as well as pathogen defense-related gene expression.

| **Target Gene** | **Primer**  **Name** | **Nucleotide Sequence (5**′ **-------→ 3′)** | **Product Size (bp)** | **Reference** |
| --- | --- | --- | --- | --- |
| 16S | PA**-**F  PH**-**R | AGAGTTTGATCCTGGCTCAG  AAGGAGGTGATCCAGCCGCA | 1300– 1500 | (Edward et al., 1989) |
| *NifH* | Pol**-**F  Pol**-**R | TGCGAYCC-SAARGCBGACTC  ATSGCCATCATYTCRCCGGA | 360 | (Poly et al., 2001) |
| *acdS* | ACD-F  ACD-R | GCAACAAGACGCGCAAGYTNGARTAYN T  GTGCATCGACTTGCCCTCRWANACNGG RT | 750-755 | ( Li. 2011) |
| **RT- qPCR Primers** | | | | |
| *NifH* | Pol**-**F  Pol**-**R | TGCGAYCC-SAARGCBGACTC  ATSGCCATCATYTCRCCGGA | RT-qPCR | (Poly et al., 2001) |
| Glyceraldehyde 3-phosphate dehydrogenase | GAPDH-1  GAPDH-2 | CTCTGCCCCAAGCAAAGATG  TGTTGTGCAGCTAGCATTG | RT-qPCR | (Niu et al., 2015) |
| *SuCAT* | CAT-F  CAT-R | CTTGTCTGGAGCACATACACTTGGA  TTCTCCGCATAGACCTTGAACTTTG | RT-qPCR | (Chen et al., 2012) |
| *SuSOD* | SOD-F  SOD-R | TTTGTCCAAGAGGGAGATGG  CTTCTCCAGCGGTGACATTT | RT-qPCR | (Jain et al., 2015) |
| *SuPAL* | PAL-F  PAL-R | CTCGAGGAGAACATCAAGAC  GTGATGAGCTCCTTCTCG | RT-qPCR | (Song et al., 2013) |
| *SuCHI* | ScChi-QF  ScChi-QR | ACGGCTACGGCGACAACA  GTCCGCTGACCAGATGAAGAG | RT-qPCR | (Su et al., 2014) |
| *SuGLU* | D-QF  D-QR | TGCTACTTCTTATCCACCCTCTG  CGTTGACATAGAAAGGTGAGCC | RT-qPCR | (Su et al., 2013) |

**REFERENCES**

Edwards, U., Rogall, T., Blöcker, H., Emde, M., and Böttger, E. C. (1989). Isolation and direct complete nucleotide determination of entire genes. Characterization of a gene coding for 16S ribosomal RNA. *Nucleic Acids Res*. 17, 7843–7853.

Poly, F., Monrozier, L.J., and Bally, R. (2001). Improvement in the RFLP procedure for studying the diversity of *nifH* genes in communities of nitrogen fixers in soil. *Res. Microbiol.* 152, 95-103.

Li, Z., Chang, S., Lin, L., Li., Y., and An, Q. (2011). A colorimetric assay of 1- aminocyclopropane-1-carboxylate (ACC) based on ninhydrin reaction for rapid screening of bacteria containing ACC deaminase. *Lett Appl Microbiol.*  53, 178-85.

Niu, J. Q., Wang, A. Q., Huang, J. L., Li, Y. R., and Yang, L. T. (2013). Cloning and expression analysis of a soluble acid invertase gene (SoSAI1) of sugarcane. *Sci. Agric. Sin*. 46, 5248–5260. doi: 10.3864/j.issn.0578-1752.2013. 24.019

Song, X. P., Huang, X., Mo, F. L., Tian, D. D., Yang, L. T., Li, Y. R., and Chen, B. S. (2013). Cloning and expression analysis of sugarcane phenylalanine ammonia-lyase (PAL) Gene. *Sci. Agric. Sin*. 46, 2856-2868.

Chen, H. J., Wu, S. D., Huang, G. J., Shen, C. Y., Afiyanti, M., Li, W. J., and Lin, Y. H. (2012). Expression of a cloned sweet potato catalase SPCAT1 alleviates ethephon-mediated leaf senescence and H_2_O_2_ elevation. *J. Plant Physiol*. 169, 86-97.

Jain, R., Chandra, A., Venugopalan, V. K., and Solomon, S. (2015). Physiological changes and expression of SOD and P5CS genes in response to water deficit in sugarcane. *Sugar Tech*. 17, 276-282.

Su, Y., Guo, J., Ling, H., Chen, S., Wang, S., Xu, L., Allan, A. C., and Que, Y. (2014). Isolation of a novel peroxisomal catalase gene from sugarcane, which is responsive to biotic and abiotic stresses. *PLoS ONE*. 9(1), e84426.

Su, Y. C., Xu, L. P., Xue, B. T., Wu, Q. B., Guo, J. L., Wu, L. G., and Que, Y. X. (2013). Molecular cloning 840 and characterization of two pathogenesis related ß-1,3-glucanase genes ScGluA1 and ScGluD1841 from sugarcane infected by *Sporisorium scitamineum*. *Plant Cell Reports*. 32, 1503-1519.

**Table S3** List of carbon, nitrogen, osmolytes, and pH present in each well of GENIII, PM3B, PM9, and PM10 BIOLOG plates.

| **S. No.** | **Serial No.** | **Carbon** | **Nittrogen** | **Osmolyte** | **pH** |
| --- | --- | --- | --- | --- | --- |
| 1 | A1 | Negative Control | Negative Control | NaCl 1% | pH 3.5 |
| 2 | A2 | Dextrin | Ammonia | NaCl 2% | pH 4 |
| 3 | A3 | D-Maltose | Nitrite | NaCl 3% | pH 4.5 |
| 4 | A4 | D-Trehalose | Nitrate | NaCl 4% | pH 5 |
| 5 | A5 | D-Cellobiose | Urea | NaCl 5% | pH 5.5 |
| 6 | A6 | Gentiobiose | Biuret | NaCl 5.5% | pH 6 |
| 7 | A7 | Sucrose | L -Alanine | NaCl 6% | pH 7 |
| 8 | A8 | D-Turanose | L-Arginine | NaCl 6.5% | pH 8 |
| 9 | A9 | Stachyose | L-Asparagine | NaCl 7% | pH 8.5 |
| 10 | A10 | Positive Control | L - Aspartic Acid | NaCl 8% | pH 9 |
| 11 | A11 | pH 6 | L-Cysteine | NaCl 9% | pH 9.5 |
| 12 | A12 | pH 5 | L-Glutamic Acid | NaCl 10% | pH 10 |
| 13 | B1 | D-Raffinose | L-Glutamine | NaCl 6% | pH 4.5 |
| 14 | B2 | α-D-Lactose | Glycine | NaCl 6% + Betaine | pH 4.5 + L-Alanine |
| 15 | B3 | D-Melibiose | L-Histidine | NaCl 6% +N-N Dimethyl glycine | pH 4.5 + L-Arginine |
| 16 | B4 | β-Methyl-D-Glucoside | L-Isoleucine | NaCl 6% + Sarcosine | pH 4.5 + L- Asparagine |
| 17 | B5 | D-Salicin | L-Leucine | NaCl 6% + Dimethyl sulphonyl propionate | pH 4.5 + L- Aspartic Acid |
| 18 | B6 | N-Acetyl-D-Glucosamine | L-Lysine | NaCl 6% + MOPS | pH 4.5 + L- Glutamic Acid |
| 19 | B7 | N-Acetyl-β-DMannosamine | L-Methionine | NaCl 6% + Ectoine | pH 4.5 + L-Glutamine |
| 20 | B8 | N-Acetyl-D-Galactosamine | L- Phenylalanine | NaCl 6% + Choline | pH 4.5 + Glycine |
| 21 | B9 | N-AcetylNeuraminic Acid | L-Proline | NaCl 6% + Phosphoryl choline | pH 4.5 + L-Histidine |
| 22 | B10 | 1% NaCl | L-Serine | NaCl 6% + Creatine | pH 4.5 + L-Isoleucine |
| 23 | B11 | 4% NaCl | L-Threonine | NaCl 6% + Creatinine | pH 4.5 + L-Leucine |
| 24 | B12 | 8% NaCl | L-Tryptophan | NaCl 6% + L - Carnitine | pH 4.5 + L-Lysine |
| 25 | C1 | α-D-Glucose | L-Tyrosine | NaCl 6% + KCl | pH 4.5 + L-Methionine |
| 26 | C2 | D-Mannose | L -Valine | NaCl 6% + L-proline | pH 4.5 + L-Phenylalanine |
| 27 | C3 | D-Fructose | D-Alanine | NaCl 6% + N -Acethyl L-glutamine | pH 4.5 + L-Proline |
| 28 | C4 | D-Galactose | D-Asparagine | NaC1 6% + β-Glutamic acid | pH 4.5 + L-Serine |
| 29 | C5 | 3-Methyl Glucose | D-Aspartic Acid | NaC1 6% + γ –Amino -n -butyric acid | pH 4.5 + L-Threonine |
| 30 | C6 | D-Fucose | D-Glutamic Acid | NaC1 6% + Glutathione | pH 4.5 + L-Tryptophan |
| 31 | C7 | L-Fucose | D-Lysine | NaCl 6% + Glycerol | pH 4.5 + L-Tyrosine |
| 32 | C8 | L-Rhamnose | D-Serine | NaC1 6% + Trehalose | pH 4.5 + L-Valine |
| 33 | C9 | Inosine | D-Valine | NaC1 6% + Trimethylamine -N-oxide | pH 4.5 + Hydroxy- L-Proline |
| 34 | C10 | 1% Sodium Lactate | L-Citrulline | NaC1 6% + Trimethylamine | pH 4.5 + L-Ornithine |
| 35 | C11 | Fusidic Acid | L-Homoserine | NaCl 6% + Octopine | pH 4.5 + L-Homoarginine |
| 36 | C12 | D-Serine | L-Ornithine | NaC1 6% + Trigonelline | pH 4.5 + L-Homoserine |
| 37 | D1 | D-Sorbitol | N - Acetyl-D, L-Glutamic Acid | Potassium chloride 3% | pH 4.5 + Anthranilic acid |
| 38 | D2 | D-Mannitol | N-Phthaloyl-L Glutamic Acid | Potassium chloride 4% | pH 4.5 + L-Norleucine |
| 39 | D3 | D-Arabitol | L-Pyroglutamic Acid | Potassium chloride 5% | pH 4.5 + L-Norvaline |
| 40 | D4 | myo-Inositol | Hydroxylamine | Potassium chloride 6% | pH 4.5 + L-α- Amino - N- butyric acid |
| 41 | D5 | Glycerol | Methylamine | Sodium sulfate 2% | pH 4.5 + L- p - Aminobenzoate |
| 42 | D6 | D-Glucose-6-PO_4_ | N-Amylamine | Sodium sulfate 3% | pH 4.5 + L- Cysteic acid |
| 43 | D7 | D-Fructose-6-PO_4_ | N-Butylamine | Sodium sulfate 4% | pH 4.5 + D-Lysine |
| 44 | D8 | D-Aspartic Acid | Ethylamine | Sodium sulfate 5% | pH 4.5 + 5-Hydroxy Lysine |
| 45 | D9 | D-Serine | Ethanolamine | Ethylene glycol 5% | pH 4.5 + 5-Hydroxy Tryptophan |
| 46 | D10 | Troleandomycin | Ethylenediamine | Ethylene glycol 10% | pH 4.5 + D, L-Diamino pimelic acid |
| 47 | D11 | Rifamycin SV | Putrescine | Ethylene glycol 15% | pH 4.5 + Trimethyl amine-N-oxide |
| 48 | D12 | Minocycline | Agmatine | Ethylene glycol 20% | pH 4.5 + Urea |
| 49 | E1 | Gelatin | Histamine | Sodium formate 1% | pH 9.5 |
| 50 | E2 | Glycyl-L-Proline | β-Phenylethyl-amine | Sodium formate 2% | pH 9.5 + L-Alanine |
| 51 | E3 | L-Alanine | Tyramine | Sodium formate 3% | pH 9.5 + L-Arginine |
| 52 | E4 | L-Arginine | Acetamide | Sodium formate 4% | pH 9.5 + L-Asparagine |
| 53 | E5 | L-Aspartic Acid | Formamide | Sodium formate 5% | pH 9.5 + L-Aspartic Acid |
| 54 | E6 | L-Glutamic Acid | Glucuronamide | Sodium formate 6% | pH 9.5 + L-Glutamic Acid |
| 55 | E7 | L-Histidine | D, L-Lactamide | Urea 2% | pH 9.5 + L-Glutamine |
| 56 | E8 | L-Pyroglutamic Acid | D-Glucosamine | Urea 3% | pH 9.5 + Glycine |
| 57 | E9 | L-Serine | D-Galactosamine | Urea 4% | pH 9.5 + L-Histidine |
| 58 | E10 | Lincomycin | D-Mannosamine | Urea 5% | pH 9.5 + L-Isoleucine |
| 59 | E11 | Guanidine HCl | N-Acetyl-D-Glucosamine | Urea 6% | pH 9.5 + L-Leucine |
| 60 | E12 | Niaproof 4 | N-Acetyl-D-Galactosamine | Urea 7% | pH 9.5 + L-Lysine |
| 61 | F1 | Pectin | N-Acetyl-D-Mannosamine | Sodium Lactate 1% | pH 9.5 + L-Methionine |
| 62 | F2 | D-Galacturonic Acid | Adenine | Sodium Lactate 2% | pH 9.5 + L-Phenylalanine |
| 63 | F3 | L-Galactonic Acid Lactone | Adenosine | Sodium Lactate 3% | pH 9.5 + L-Proline |
| 64 | F4 | D-Gluconic Acid | Cytidine | Sodium Lactate 4% | pH 9.5 + L-Serine |
| 65 | F5 | D-Glucuronic Acid | Cytosine | Sodium Lactate 5% | pH 9.5 + L-Threonine |
| 66 | F6 | Glucuronamide | Guanine | Sodium Lactate 6% | pH 9.5 + L-Tryptophan |
| 67 | F7 | Mucic Acid | Guanosine | Sodium Lactate 7% | pH 9.5 + L-Tyrosine |
| 68 | F8 | Quinic Acid | Thymine | Sodium Lactate 8% | pH 9.5 + L-Valine |
| 69 | F9 | D-Saccharic Acid | Thymidine | Sodium Lactate 9% | pH 9.5 + Hydroxy- L-Proline |
| 70 | F10 | Vancomycin | Uracil | Sodium Lactate 10% | pH 9.5 + L-Ornithine |
| 71 | F11 | Tetrazolium Violet | Uridine | Sodium Lactate 11% | pH 9.5 + L-Homoarginine |
| 72 | F12 | Tetrazolium Blue | Inosine | Sodium Lactate 12% | pH 9.5 + L-Homoserine |
| 73 | G1 | p-Hydroxy- Phenylacetic Acid | Xanthine | Sodium Phosphate pH 7 20mM | pH 9.5 + Anthranilic acid |
| 74 | G2 | Methyl Pyruvate | Xanthosine | Sodium Phosphate pH 7 50mM | pH 9.5 + L-Norleucine |
| 75 | G3 | D-Lactic Acid Methyl Ester | Uric Acid | Sodium Phosphate pH 7 100mM | pH 9.5 + L-Norvaline |
| 76 | G4 | L-Lactic Acid | Alloxan | Sodium Phosphate pH 7 200mM | pH 9.5 + Agmatine |
| 77 | G5 | Citric Acid | Allantoin | Sodium Benzoate pH 5.2 20mM | pH 9.5 + Cadaverine |
| 78 | G6 | α-Keto-Glutaric Acid | Parabanic Acid | Sodium Benzoate pH 5.2 50mM | pH 9.5 + Putrescine |
| 79 | G7 | D-Malic Acid | D, L-α-Amino-N-Butyric Acid | Sodium Benzoate pH 5.2 100mM | pH 9.5 + Histamine |
| 80 | G8 | L-Malic Acid | γ-Amino-N-Butyric Acid | Sodium Benzoate pH 5.2 200mM | pH 9.5 + Phenylethylamine |
| 81 | G9 | Bromo-Succinic Acid | ε-Amino-N-Caproic Acid | Ammonium sulfate pH8 10mM | pH 9.5 + Tyramine |
| 82 | G10 | Nalidixic Acid | D, L-α-Amino-Caprylic Acid | Ammonium sulfate pH8 20mM | pH 9.5 + Creatine |
| 83 | G11 | Lithium Chloride | δ-Amino-N-Valeric Acid | Ammonium sulfate pH8 50mM | pH 9.5 + Trimethyl amine- N-oxide |
| 84 | G12 | Potassium Tellurite | α-Amino-N-Valeric Acid | Ammonium sulfate pH8 100mM | pH 9.5 + Urea |
| 85 | H1 | Tween 40 | Ala-Asp | Sodium Nitrate 10mM | X-Caprylate |
| 86 | H2 | γ-Amino-ButryricAcid | Ala-Gln | Sodium Nitrate 20mM | X–α-D-Glucoside |
| 87 | H3 | α-Hydroxy- Butyric Acid | Ala-Glu | Sodium Nitrate 40mM | X-β-D-Glucoside |
| 88 | H4 | β-Hydroxy-D,LButyricAcid | Ala-Gly | Sodium Nitrate 60mM | X-α-D-Galactoside |
| 89 | H5 | α-Keto-Butyric Acid | Ala-His | Sodium Nitrate 80mM | X-β-D-Galactoside |
| 90 | H6 | Acetoacetic Acid | Ala-Leu | Sodium Nitrate 100mM | X-α-D-Glucuronide |
| 91 | H7 | Propionic Acid | Ala-Thr | Sodium Nitrite 10mM | X-β-D-Glucuronide |
| 92 | H8 | Acetic Acid | Gly-Asn | Sodium Nitrite 20mM | X-β-D-Glucosaminide |
| 93 | H9 | Formic Acid | Gly-Gln | Sodium Nitrite 40mM | X-β-D-Galactosaminide |
| 94 | H10 | Aztreonam | Gly-Glu | Sodium Nitrite 60mM | X-α-D-Mannoside |
| 95 | H11 | Sodium Butyrate | Gly-Met | Sodium Nitrite 80mM | X-PO4 |
| 96 | H12 | Sodium Bromate | Met-Ala | Sodium Nitrite 100mM | X-SO4 |

**TABLE S4** Physicochemical characterization of rhizospheric soils used for the isolation of rhizobacteria collected from the sugarcane fields.

| **Parameters** | **Rhizosphere Soil** | | | | |
| --- | --- | --- | --- | --- | --- |
|  | | **GT11** | **GXB9** | **GT29** | **ROC22** |
| **Primary macronutrients (g kg^-1^)** | | | | | |
| Total N | | 0.34±0.01^c^ | 0.40±0.01^b^ | 0.43±0.01^b^ | 1.23±0.02^a^ |
| Available NO_3_^-^N | | 14.60±0.22^a^ | 8.20±0.12^b^ | 7.18±0.11^c^ | 5.74±0.09^d^ |
| Available NH_4_^+^N | | 3.13±0.05^d^ | 4.73±0.07^c^ | 6.64±0.10^b^ | 9.79±0.15^a^ |
| Total P | | 0.43±.01^b^ | 0.46±0.01^a^ | 0.40±0.01^c^ | 0.41±0.01^bc^ |
| Available-P (mg kg^-1^) | | 185.80±2.80^a^ | 64.70±0.0.97^c^ | 53.20±0.80^d^ | 79.30±1.19^b^ |
| Total K | | 13.29±0.20^b^ | 13.34±0.20^b^ | 14.37±0.22^a^ | 14.26±0.21^a^ |
| Available-K (mg kg^-1^) | | 141.00±2.12^a^ | 64.00±0.96^c^ | 71.00±1.07^b^ | 35.00±0.53^d^ |
| **Secondary macronutrients (mg kg^-1^)** | | | | | |
| Calcium | | 813.63±12.25^a^ | 749.15±11.28^b^ | 800.10±12.05^a^ | 775.90±11.69^ab^ |
| Magnesium | | 147.92±2.23^a^ | 152.46±2.30^a^ | 152.21±2.29^a^ | 137.86±2.08^b^ |
| **Micronutrients (mg kg^-1^)** | | | | | |
| Iron (Fe) | | 150.31±2.26^a^ | 120.71±1.82^b^ | 91.01±1.37^c^ | 62.54±0.94^d^ |
| Manganese (Mn) | | 88.46±1.33^a^ | 80.21±1.21^b^ | 84.08±1.27^b^ | 89.56±1.35^a^ |
| Zinc (Zn) | | 8.17±0.12^a^ | 7.49±0.11^b^ | 7.26±0.11^b^ | 6.79±0.10^c^ |
| Boron (B) | | 0.44±0.01^b^ | 0.39±0.01^c^ | 0.39±0.01^c^ | 0.49±0.01^a^ |
| Sulfate (SO_4_^2-^) | | 144.14±2.17^a^ | 132.66±2.00^b^ | 144.47±2.18^a^ | 131.67±1.98^b^ |
| Chlorine (Cl^-^) | | 35.41±0.53^b^ | 38.49±0.58^a^ | 23.97±0.36^c^ | 24.70±0.37^c^ |
| **Physical properties** | | | | | |
| Sand (2.0-0.05 mm) (g kg^-1^) | | 177.23±2.67^c^ | 162.15±2.44^d^ | 207.25±3.12^b^ | 217.32±3.27^a^ |
| Silt (2.0-0.05 mm) (g kg^-1^) | | 310.05±4.67^c^ | 328.14±4.94^b^ | 346.51±5.22^a^ | 355.28±5.35^a^ |
| Clay (2.0-0.05 mm) (g kg^-1^) | | 225.18±3.39^b^ | 205.26±3.09^c^ | 238.88±3.60^a^ | 228.26±3.44^b^ |
| pH | | 6.70±0.10^a^ | 6.22±0.09^b^ | 6.09±0.09^b^ | 5.99±0.09^b^ |
| EC (Sm^−1^) | | 0.0113^a^ | 0.00712^a^ | 0.00811^a^ | 0.00829^a^ |
| Water Content (%) | | 5.43±0.08^b^ | 6.18±0.09^a^ | 6.11±0.09^a^ | 5.13±0.08^c^ |
| Texture | | Medium loam | Medium loam | Medium loam | Medium loam |
| Color | | Pale red | Pale red | Pale red | Pale red |

Mean values not sharing a common letter differ significantly (*P*<0.05) from each other.

**Table S5** Identification of nitrogen-fixing bacteria from sugarcane soil based on the 16S rRNA gene sequence.

| **Culture Code** | **Identification** | **% Similarity** | **Amplicon Size (bp)** | **Accessions Number Match** |
| --- | --- | --- | --- | --- |
| AA1 | *Pantoea* sp. | 100 | 1397 | LC484790 |
| AA2 | *Pantoea agglomerans* | 100 | 1394 | HQ443233 |
| AA3 | *Enterobacter ludwigii* | 100 | 1403 | MN208236 |
| AA7 | *Pantoea dispersa* | 100 | 1402 | KM019829 |
| AA10 | *Enterobacter cloacae* | 99.31 | 1473 | MN853571 |
| AN3 | *Enterobacter* sp. | 100 | 1404 | MN560062 |
| AN4 | *Enterobacter ludwigii* | 98.84 | 1403 | MN181133 |
| AY3 | *Enterobacter aerogenes* | 100 | 1400 | KU500561 |
| AY5 | *Enterobacter huaxiensis* | 99.86 | 1440 | MK734322 |
| BA18 | *Enterobacter asburiae* | 99.85 | 1342 | MH200614 |
| BN4 | *Enterobacter* sp. | 99.71 | 1438 | JN210900 |
| BY1 | *Erwinia* sp. | 100 | 1350 | KU891828 |
| BY4 | *Enterobacter asburiae* | 99.86 | 1437 | HQ242719 |
| BY5 | *Pantoea dispersa* | 99.96 | 1343 | MN833616 |
| CA2 | *Enterobacter tabaci* | 100 | 1342 | MF682952 |
| CA3 | *Enterobacter cancerogenus* | 99.47 | 1340 | MN177191 |
| CA9 | *Enterobacter cloacae* | 99.77 | 1337 | KF516281 |
| CA11 | *Enterobacter aerogenes* | 99.46 | 1328 | JF431274 |
| CN8 | *Enterobacter mori* | 99.69 | 1340 | MK11464 |
| CN18 | *Enterobacter asburiae* | 99.62 | 1342 | HQ242719 |
| CN19 | *Enterobacter cancerogenus* | 99.92 | 1333 | KM019851 |
| CoA3 | *Pantoea dispersa* | 99.71 | 1349 | MK156738 |
| CoA8 | *Enterobacter oryzae* | 99.87 | 1499 | JF513179 |
| CoA9 | *Pantoea agglomerans* | 99.72 | 1440 | HM130695 |
| CoA11 | *Pantoea* sp. | 100 | 1453 | KJ184998 |
| CoA12 | *Erwinia* sp. | 99.86 | 1461 | KJ184863 |
| CoN2 | *Enterobacter sacchari* | 100 | 1513 | HQ204314 |

**TABLE S6** The rhizospheric microbial activity calculated based on color development and important functional diversity indices at 72 h of incubation for 95 different sole carbon, nitrogen, osmolytes, and pH of BIOLOG Phenotype Micro-Array^TM^ plates.

| **Diversity Parameters** | ***Enterobacter asburiae* (BY4)** | | | | ***Pantoea dispersa* (AA7)** | | | |
| --- | --- | --- | --- | --- | --- | --- | --- | --- |
|  | Carbon | Nitrogen | Osmolytes | pH | Carbon | Nitrogen | Osmolytes | pH |
|  | GENIII | PM3B | PM9 | PM10 | GENIII | PM3B | PM9 | PM10 |
| Simpson_1-D | 0.9886 | 0.9873 | 0.9435 | 0.9564 | 0.9852 | 0.9849 | 0.9829 | 0.9884 |
| Shannon_H | 4.530 | 4.492 | 3.935 | 3.944 | 4.345 | 4.326 | 4.166 | 4.477 |
| Evenness_e^H/S | 0.9658 | 0.9303 | 0.5331 | 0.5376 | 0.803 | 0.7877 | 0.6714 | 0.9163 |
| Brillouin | 0.4596 | 0.4912 | 0.3998 | 0.3475 | 1.112 | 1.164 | 1.654 | 2.099 |
| Menhinick | 26.020 | 32.270 | 33.220 | 28.140 | 11.090 | 11.310 | 9.786 | 7.566 |
| Equitability_J | 0.9924 | 0.9842 | 0.8622 | 0.864 | 0.9519 | 0.9477 | 0.9127 | 0.9808 |

**TABLE S7** Expression analysis of *nifH* gene in leaf tissues of sugarcane varieties (GT11 and GXB9) inoculated with *P. dispersa* (AA7) and *E. asburiae* (BY4) by qRT-PCR.

| **Treatment** | **Days** | **GT11** | **GXB8** |
| --- | --- | --- | --- |
| BY4 | 30 | 5.64±0.217^a^ | 2.89±0.079^a^ |
| AA7 | 30 | 3.05±0.096^b^ | 1.56±0.054^c^ |
| BY4 | 60 | 1.62±0.060^c^ | 1.80±0.077^b^ |
| AA7 | 60 | 1.05±0.046^d^ | 1.06±0.049^d^ |

**TABLE S8** The effect of *P. dispersa* (AA7) and *E. asburiae* (BY4) strains on the relative gene expression levels of stress-related genes in GT11 and GXB9 sugarcane varieties.

| **Treatment** | **Days** | **GT11** | **GXB9** | **GT11** | **GXB9** | **GT11** | **GXB9** | **GT11** | **GXB9** | **GT11** | **GXB9** |
| --- | --- | --- | --- | --- | --- | --- | --- | --- | --- | --- | --- |
|  |  | *SuSOD* | *SuSOD* | *SuCAT* | *SuCAT* | *SuPAL* | *SuPAL* | *SuCHI* | *SuCHI* | *SuGLU* | *SuGLU* |
| BY4 | 30 | 1.24±0.038^d^ | 1.84±0.04^c^ | 1.74±0.044^c^ | 2.48±0.047^c^ | 2.43±0.04^c^ | 3.79± 0.056^c^ | 2.76±0.046^c^ | 3.44±0.051^c^ | 3.20±0.057^b^ | 3.29±0.059^c^ |
| AA7 | 30 | 1.39±0.051^c^ | 1.56±0.03^c^ | 1.66±0.035^c^ | 2.22±0.043^d^ | 2.15±0.032^d^ | 2.50±0.047^d^ | 2.32±0.050^d^ | 2.82±0.042^d^ | 2.12±0 .041^c^ | 2.24±0 .033^d^ |
| BY4 | 60 | 2.67±0.040^b^ | 3.35±0.06^a^ | 3.13±0 .046^a^ | 3.59±0 .063^a^ | 5.15±0.086^a^ | 5.58±0.093^a^ | 5.55±0.092^a^ | 6.14±0 .096^b^ | 4.45±0 .066^a^ | 4.81±0.071^a^ |
| AA7 | 60 | 2.89±0.063^a^ | 3.20±0.05^b^ | 2.85±0.042^b^ | 3.15±0.057^b^ | 4.47±0.066^b^ | 5.29±0.078^b^ | 4.49±0.067^b^ | 6.38±0.115^a^ | 3.26±0 .054^b^ | 3.93±0.058^b^ |

**TABLE S9** Biological nitrogen fixation estimation by rhizobacteria *P. dispersa* (AA7) and *E. asburiae* (BY4) in GT11 and GXB9 sugarcane plant tissues at 180 days after inoculation.

1. **Total N%**

| **GT11** | **Treatment** | **Leaf** | **Root** | **Stem** |
| --- | --- | --- | --- | --- |
|  | Control | 8.96±0.365^c^ | 14.63±0.769^c^ | 9.88±0.482^c^ |
|  | BY4 | 29.77±0.647^a^ | 35.90±01.26^a^ | 17.69±0.625^a^ |
|  | AA7 | 18.88±0.456^b^ | 21.09±0.988^b^ | 15.33±0.582^b^ |

| **GXB9** | **Treatment** | **Leaf** | **Root** | **Stem** |
| --- | --- | --- | --- | --- |
|  | Control | 9.91±0.349^c^ | 16.83±0.753^c^ | 6.20±0.293^c^ |
|  | BY4 | 10.95±0.401^b^ | 37.06±1.138^a^ | 21.53±0.996^a^ |
|  | AA7 | 14.94±0.675^a^ | 31.60±1.18^b^ | 17.30±0.718^b^ |

1. **^15^N Atom%**

| **GT11** | **Treatment** | **Leaf** | **Root** | **Stem** |
| --- | --- | --- | --- | --- |
|  | Control | 1.16±0.027^b^ | 1.31±0.026^b^ | 1.01±0.015^c^ |
|  | BY4 | 1.99±0.040^a^ | 2.22±0.043^a^ | 1.79±0.027^b^ |
|  | AA7 | 2.01±0.040^a^ | 2.22±0.043^a^ | 2.04±0.031^a^ |

| **GXB9** | **Treatment** | **Leaf** | **Root** | **Stem** |
| --- | --- | --- | --- | --- |
|  | Control | 1.19±0.028^c^ | 1.27±0.019^c^ | 1.02±0.025^c^ |
|  | BY4 | 1.74±0.036^b^ | 1.86±0.038^b^ | 1.40±0.031^b^ |
|  | AA7 | 1.98±0.049^a^ | 2.16±0.042^a^ | 1.91±0.039^a^ |

## FIGURES


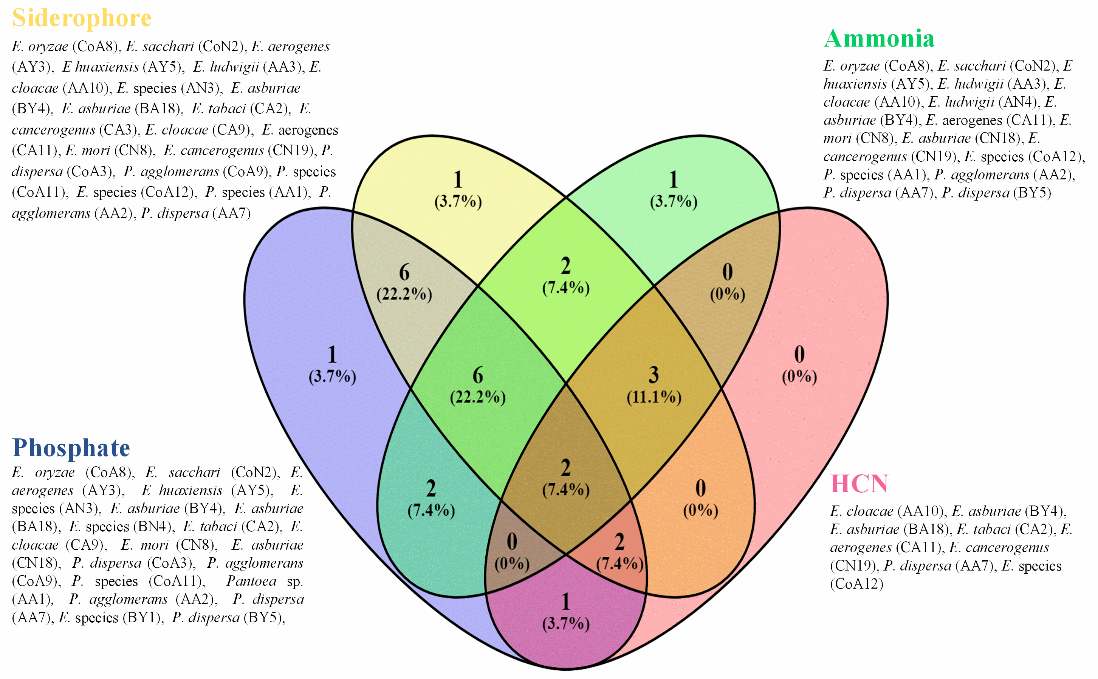


**FIGURE S1** The VENN diagram represents the number of shared and single PGP traits by rhizobacterial strains. Different colors represent different PGP activities.


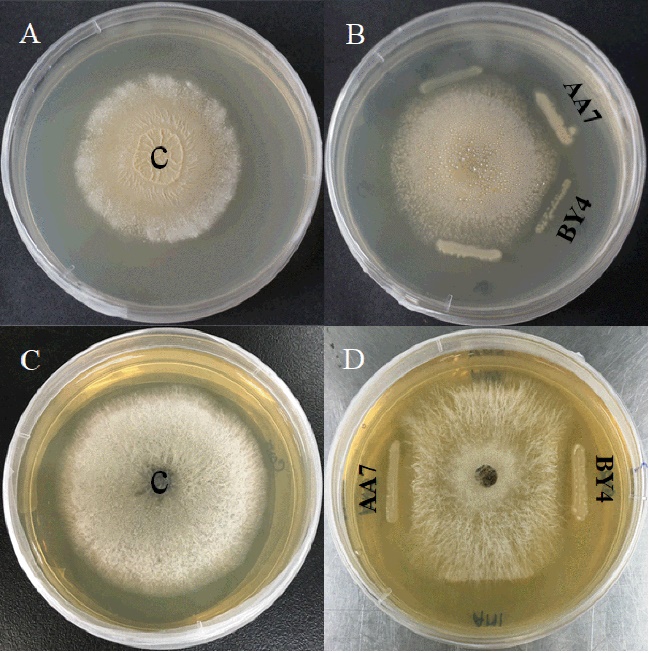


**FIGURE S2** Dual culture plate assay of selected strains (AA7 and BY4) against sugarcane pathogens. (A) *Sporisorium scitamineum* (Control), (B) Antifungal activity of strains against *S. scitamineum*, (C) *Ceratocystis paradoxa* (Control), and (D) Antifungal activity of strains against *C. paradoxa*





**FIGURE S3** The *acdS* gene amplification of rhizobacterial strains, around 755 bp fragments to be amplified. M is a molecular size marker (100 to 2,000 bp), PC is positive control (*Pseudomonas entomophila*), and NC is negative control (sterile water).


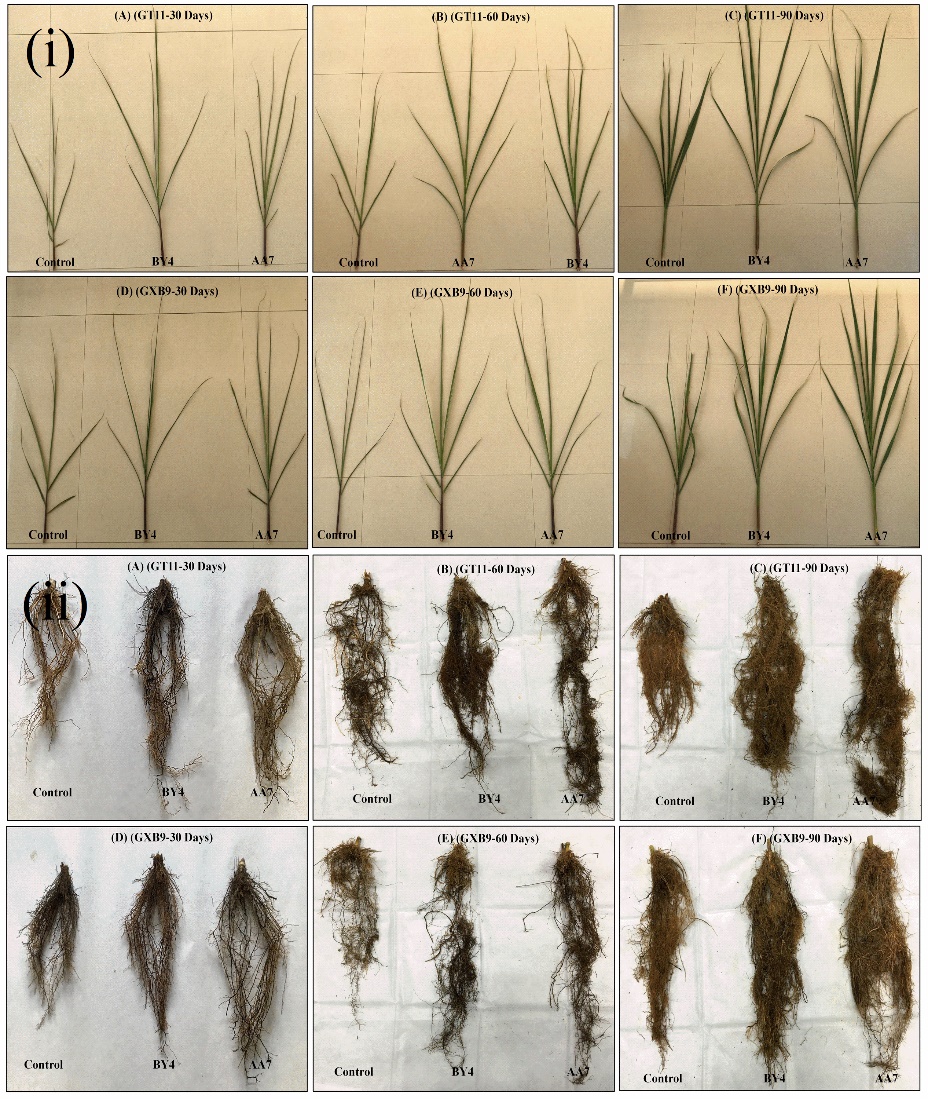


**FIGURE S4** Sugarcane (GT11 and GXB9) growth promotion by DPGPR, *P. dispersa* (AA7) and *E. asburiae* (BY4) at 30- 60- and 90-days after inoculation as compared to control in the greenhouse (i) Shoot and (ii) Root development.
